# Involvement of cecropin B in the formation of the *Aedes aegypti* mosquito cuticle

**DOI:** 10.1038/s41598-017-16625-6

**Published:** 2017-11-27

**Authors:** Wei-Ting Liu, Wu-Chun Tu, Chao-Hsiung Lin, Ueng-Cheng Yang, Cheng-Chen Chen

**Affiliations:** 1 Institute of Microbiology and Immunology, National Yang-Ming University, Taipei, 112 Taiwan, ROC; 2 Department of Entomology, National Chung Hsing University, Taichung, 402 Taiwan, ROC; 3 Department of Life Sciences and Institute of Genome Sciences, National Yang-Ming University, Taipei, 112 Taiwan, ROC; 4 Institute of Biomedical Informatics, National Yang-Ming University, National Yang-Ming University, Taipei, 112 Taiwan, ROC

## Abstract

In this study, we found a mosquito antimicrobial peptide (AMP), *Aedes aegypti* cecropin B (Aacec B), was expressed constitutively in pupae. Knockdown in the pupae of Aacec B using double-stranded RNA (dsRNA) resulted in high mortality, the emergence of deformed adults and an impairment of pharate adult cuticle formation with fewer lamellae being deposited and the helicoidal pattern of the chitin microfibrils being disorganized. Simultaneous injection of Aacec B dsRNA and Aacec B peptide into pupae significantly reduced this mortality and no deformed adults then emerged. The expression levels of *Ae. aegypti* prophenoloxidase (AaPPO) 3 and AaPPO 4 were significantly reduced in the Aacec B knockdown pupae. Exogenous Aacec B peptide significantly enhanced the transcription of AaPPO 3 in pupae. Knockdown of AaPPO 3 in pupae caused effects similar to Aacec B-knockdown. The Aacec B peptide could be detected in both the cytoplasm and nuclei of pupal cells and was able to bind to the TTGG(A/C)A motif in AaPPO 3 DNA both *in vitro* and *in vivo*. These findings suggest that Aacec B plays a crucial role in pharate adult cuticle formation via the regulation of AaPPO 3 gene expression in pupae.

## Introduction

More than 150 antimicrobial peptides (AMPs) in insects have been identified or been predicted via genome sequencing^1^. AMPs, which are small proteins, can be induced rapidly and provide non-specific killing or growth inhibition of invading microbes, including bacteria, fungi, enveloped viruses and parasites^2–4^. In addition, a few AMPs have been found to play roles in some insect physiological functions. For example, knockdown of hemolin expression leads to embryonic lethality in cecropia pupae, which suggests that hemolin is necessary for embryonic development^5^.

Cecropin was the first insect AMP discovered by Boman’s research group in the 1980’s and was isolated from the cecropia moth *Hyalophora cecropia* ^6^. Recent genome searches have revealed that multiple genes encoding various cecropins are present in the genomes of a range of insects^7–10^. The genomes of *Aedes aegypti*, *Drosophila melanogaster*, and *Bombyx mori* contain ten, six and eleven genes encoding a cecropin, respectively^7,9,10^. In *Ae. aegypti*, different cecropins can be induced by different pathogens^11–17^, for example Aacec A, D, E, and G are produced in response to bacterial injection^11,12,14–16^, while the expression levels of Aacec A and N are elevated in mosquitoes infected with dengue 2 virus^13,17^.

Yang *et al*. (2011) found that within the cecropin family from *B. mori*, the most effective *B. mori* cecropin (Bmcec) genes, namely Bmcec B6 and Bmcec D, which have the strongest antimicrobial activities, also have the highest levels of induction^10^. Furthermore, other Bmcec genes, such as Bmcec E, which have the lowest levels of induced expression, have the most limited antimicrobial spectrum and the weakest antimicrobial activity. Based on this they suggested that Bmcec B6 and Bmcec D may play crucial roles in eliminating microbial infection, while the other non-major proteins, such as Bmcec E, may function as backups to the major AMPs. We hypothesize in the present study that those AMPs with no or a low level of induced expression may not only act as backups to the major AMPs, but also may play important roles in some other physiological functions. In this paper, we demonstrate that Aacec B, which is expressed constitutively in *Ae. aegypt*i adults, and the expression level of which is not affected by bacterial challenge, plays an important role in cuticle formation by the insect’s pupae.

## Results

### The expression level of Aacec B mRNA is not affected by bacterial challenges in adults, but does fluctuated in pupae, while Aacec B peptide would seem to be present as multimers in *Ae. aegypti* pupal cells

**Figure 1:**
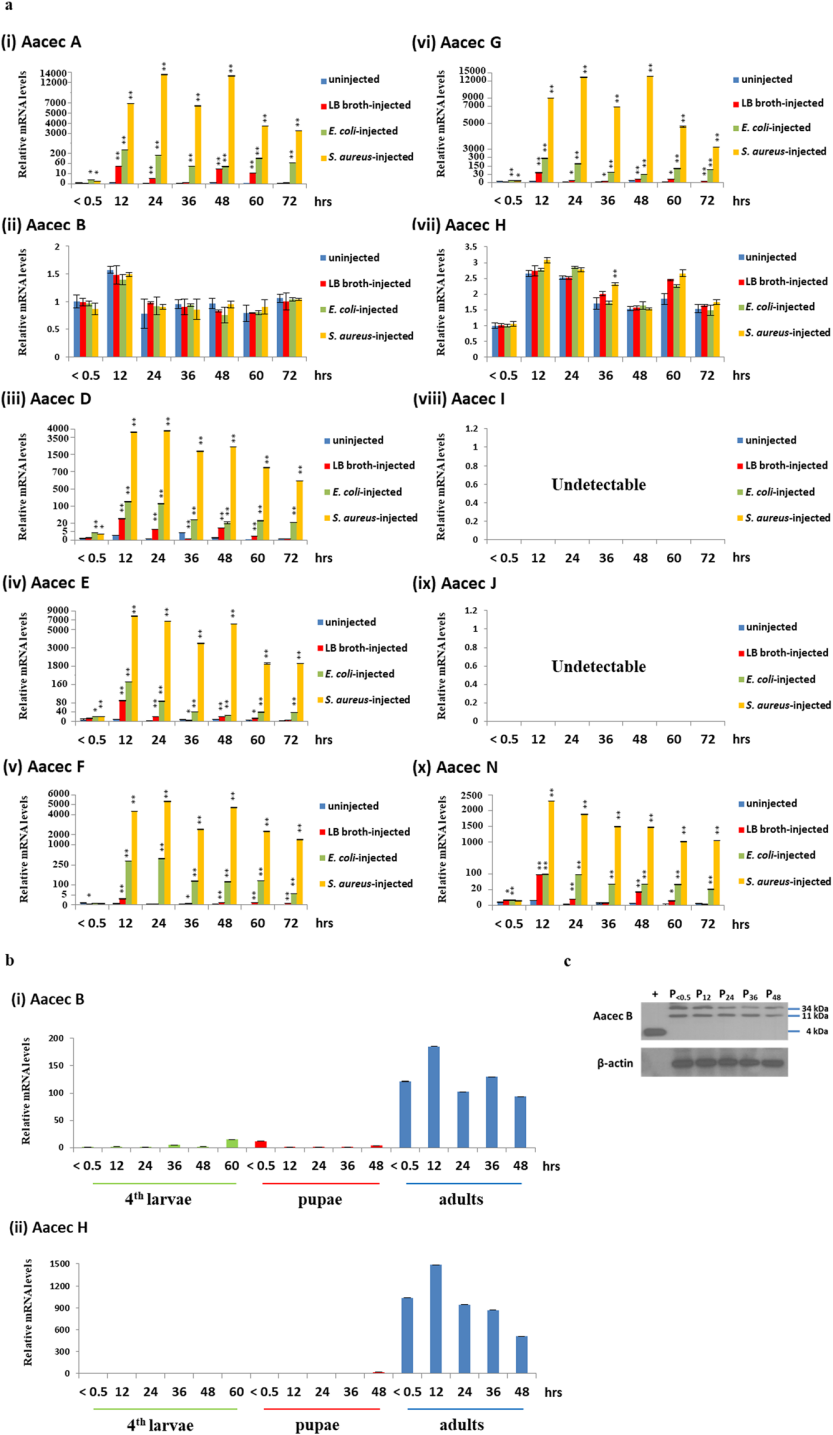
RT-qPCR analysis of the expression profiles of *Ae. aegypti* cecropins and Aacec B peptide is presented as nonameric and trimeric multimers in *Ae. aegypti* pupal cell. (**a**) The expression profiles of the *Ae. aegypti* cecropins in the uninjected control, LB broth-injected, *Escherichia coli-*injected and *Staphylococcus aureus*-injected adult mosquitoes. 0.5–72: 0.5–72 hrs after injection. The relative expression levels are expressed as means ± SD (n = 3), with the uninjected mosquitoes at <0.5 hr after injection as the calibrator. Asterisks indicate significant differences (*p < 0.005; **p < 0.001) from the relative mRNA levels of the uninjected control at each time-point. (**b**) The expression profiles of *Ae. aegypti* cecropin B and H in 4^th^ instar larvae, pupae and adults. 0.5–60: 0.5–60 hrs after ecdysis. The relative expression levels are expressed as means ± SD (n = 3), with 4^th^ instar larvae at <0.5 hr after ecdysis as the calibrator. (**c**) Western blot analysis using antibodies specific for Aacec B in total protein extract. β-actin was used as loading controls. P_< 0.5_: <0.5 hr after larval-pupal ecdysis; P_12_–P_48_: 12–48 hrs after pupation. +: synthetic Aacec B peptide. Uncropped images are shown in Supplementary Figure S1.

We first examined the expression profiles of various Aacecs in adult *Ae. aegypti* after challenge with the Gram (−) bacterium *E. coli* BL21 and Gram (+) bacterium *S. aureus* CCRC 15211. As shown in Fig. 1a, among the ten Aacecs expressed in *Ae. aegypti*, the mRNA levels of six Aacecs (Aacec A, D, E, F, G and N) were significantly induced after injection with bacteria. Furthermore, two Aacecs (Aacec I and J) could not be detected in uninjected control mosquitoes, the LB broth-injected control mosquitoes or the bacteria-injected mosquitoes. However, the two remaining Aacecs (Aacec B and H) were constitutively expressed in normal adults and their expression levels remained almost the same after bacterial challenge, respectively, except that the expression level of Aacec H was significantly increased at 36 hrs in the *S. aureus*-injected mosquitoes. Subsequently, we further examined the expression profiles of Aacec B and Aacec H in normal 4^th^ instar larvae, in pupae and in adults without bacteria challenge. As shown in Fig. 1b, in 4^th^ instar larvae, the expression of Aacec B remained low at <0.5, 12, 24 and 48 hrs after ecdysis, but it was slightly increased at 36 hrs and at 60 hrs after ecdysis. In pupae, immediately after larval-pupal ecdysis, the expression of Aacec B was detectable at <0.5 hr after ecdysis, and then decreased to low levels at 12, 24 and 36 hrs, but was again slightly increased at 48 hrs after ecdysis. It should be noted that Aacec B mRNA is highly expressed in adults. On the other hand, the expression level of Aacec H was barely detectable in both larvae and pupae, and was slightly increased to a low level at 48 hrs after larval-pupal ecdysis, and then began to be highly expressed in adults (Fig. 1b). Thus, while the mRNA of Aacec B was constitutively expressed in adults and was not affected by challenging with bacteria in adults, it did fluctuate in pupae and larvae. These results indicate that Aacec B might play important roles in pupae and larvae. Next, Western blotting was used to detect the presence of Aacec B in the pupal cells. As shown in Fig. 1c, Aacec B antibody was able to detect a single band of synthetic Aacec B peptide with an estimated molecular weight approximately 4 kDa (the theoretical molecular weight of synthetic Aacec B is 3.79 kDa). The same antibody was able to detect two bands in total protein extract from pupae. The molecular weights of these two bands were approximately 11 kDa and 34 kDa, respectively; these values correspond to those of a trimer of Aacec B and a nonamer of Aacec B. Based on the above findings, we hypothesize that Aacec B may play a physiological role in pupae.

### Knockdown of Aacec B in *Ae. aegypti* pupae leads to high pupal mortality and the emergence of deformed adults, while the effects of Aacec B knockdown are rescued by Aacec B peptide

**Figure 2:**
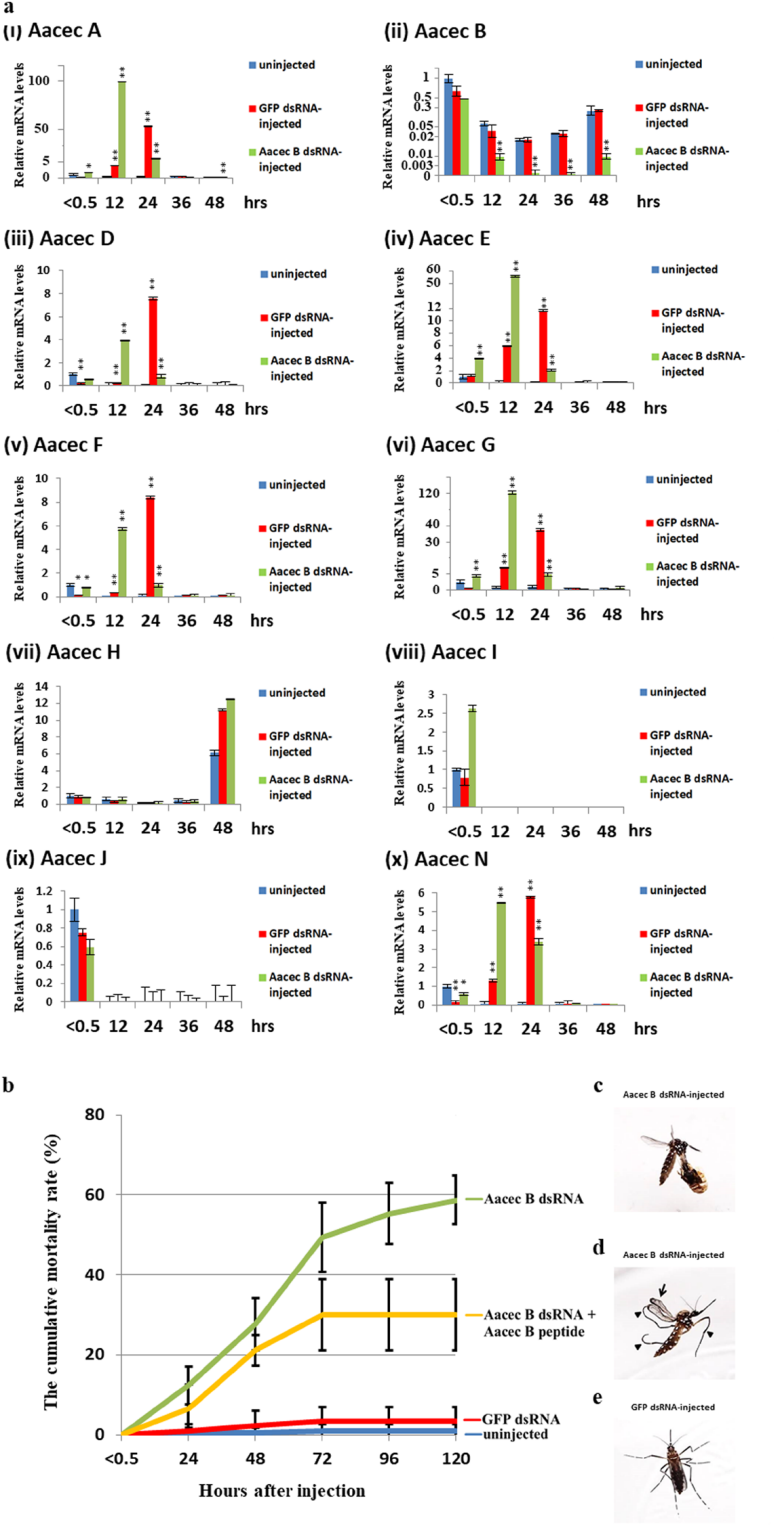
Knockdown of Aacec B in *Ae. aegypti* pupae leads to high pupal mortality and the emergence of deformed adult. (**a**) RT-qPCR analysis of *Ae. aegypti* cecropins expression in uninjected control, GFP dsRNA-injected control and Aacec B dsRNA-injected pupae. P_< 0.5_: Pupae were injected within 0.5 hr after larval-pupal ecdysis; P_12_–P_48_: 12–48 hrs after pupation. The relative expression levels are expressed as means ± SD (n = 3), with uninjected pupae at <0.5 hr after injection as the calibrator. Asterisks indicate significant differences (*p < 0.005; **p < 0.001) from the relative mRNA levels of the uninjected control at each time-point. (**b**) The cumulative mortality rates (%) of uninjected control (blue line), GFP dsRNA-injected control (red line), Aacec B dsRNA-injected (green line), and Aacec B dsRNA +Aacec B peptide-injected (orange line) *Ae. aegypti* pupae at various times after injection. P_< 0.5_: Pupae were injected within 0.5 hr after larval-pupal ecdysis; 24–120: 24–120 hrs after larval-pupal ecdysis. Each group consisted of 30 pupae in one experiment and 5 replicates were conducted. The range bars indicates the standard deviations of the means. (**c**) An adult mosquito from the Aacec B dsRNA-injected pupae that was unable to detach from the exuvia. (**d**) An adult mosquito with deformed body parts. Arrows (↑) indicate curved wings and arrow heads (▲) indicate curved legs. (**e**) A normal mosquito that emerged from the GFP dsRNA-injected pupae.

We used Aacec B double-stranded RNA (dsRNA, 277 bp, position from −58 to +219 bp) to investigate the effects of Aacec B silencing on *Ae. aegypti* pupae. Immediately after larval-pupal ecdysis, mosquito pupae were divided into two groups, one group was intrathoracically injected with Aacec B dsRNA while the other group was intrathoracically injected with GFP dsRNA (each mosquito received 1 μg dsRNA). Compared with the GFP dsRNA-injected group and the uninjected mosquitoes, the transcription level of Aacec B in Aacec B dsRNA-injected pupae was found to gradually decrease over time when measured at 12, 24, 36 and 48 hrs after larval-pupal ecdysis (Fig. 2a). However, it should be noted that six Aacecs (Aacec A, D, E, F, G and N) were significantly induced in both the GFP dsRNA-injected and Aacec B dsRNA-injected mosquitoes; this suggests that these six Aacecs might have been induced by tissue injury during injection. These results indicated that injection of Aacec B dsRNA is able to specifically knockdown the expression level of Aacec B in *Ae. aegypti* pupae. Injection with Aacec B dsRNA resulted in a high level of pupal mortality. As shown in Fig. 2b, at 120 hrs after Aacec B dsRNA injection, the cumulative mortality rate of the GFP dsRNA-injected control pupae and uninjected control pupae were 3.33 ± 3.65% and 0.95 ± 1.63%, respectively, whilst the cumulative mortality rate of the Aacec B dsRNA-injected pupae was 58.67 ± 6.13%; this included 9.34% of the Aacec B dsRNA-injected pupae that emerged as adults but were unable to detach from the pupal exuvia (Fig. 2c). About 21% of Aacec B dsRNA-injected pupae emerged as deformed adults with curved legs or wings (Fig. 2d) and died shortly after emergence. Thus only approximately 20% of the Aacec B dsRNA-injected pupae successfully emerged as normal adults. Importantly, the cumulative mortality rate (30 ± 8.82%) was significantly reduced (*p* < 0.01) and no deformed adult emerged when the pupae were injected simultaneously with 1 μg Aacec B dsRNA and 1 ng Aacec B peptide (Fig. 2b). These findings indicate that Aacec B seems to play an important role in pupal morphogenesis.

### An ultrastructural study reveals that cuticle formation was impaired in the Aacec B-knockdown pupae

**Figure 3:**
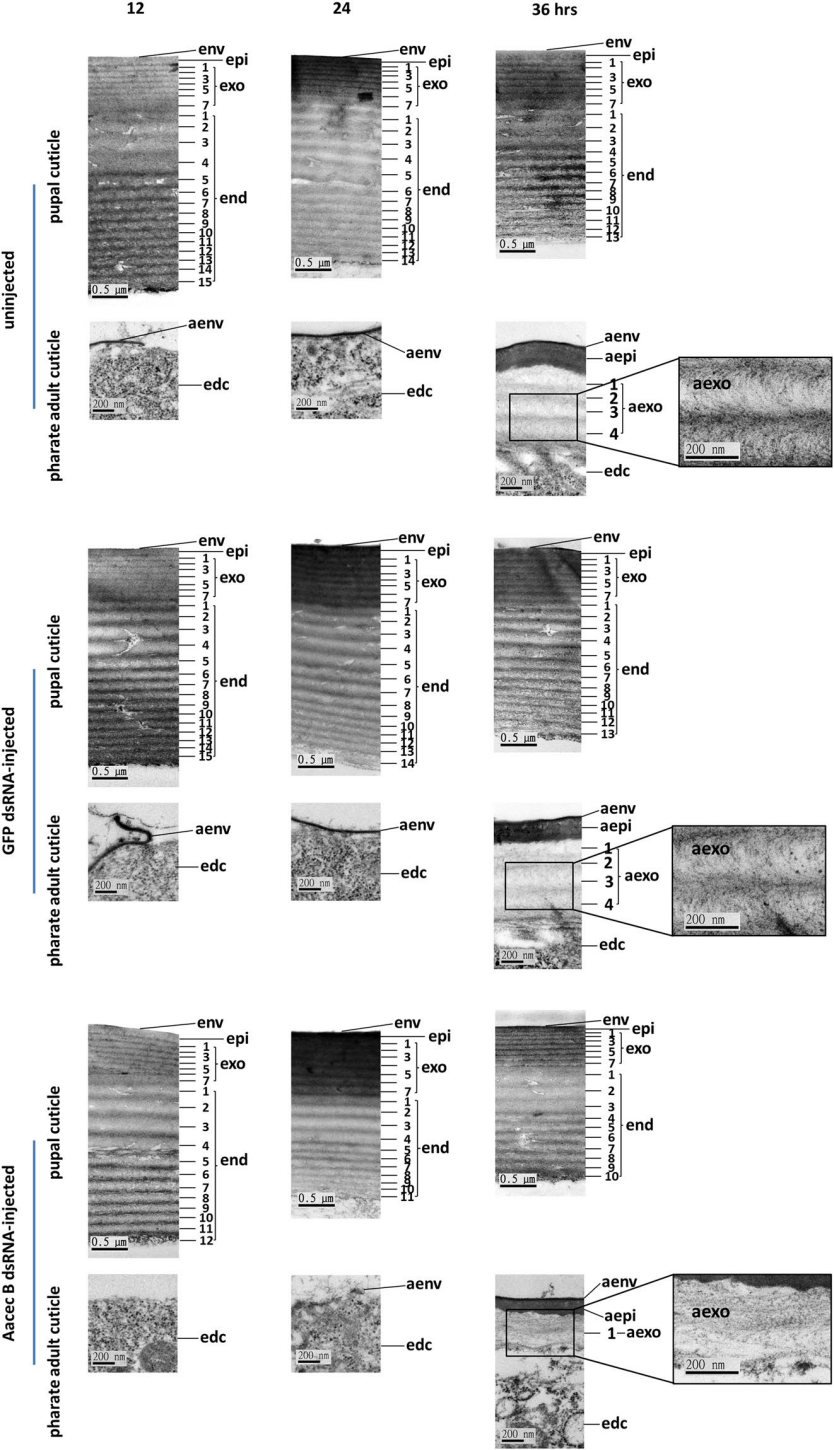
Ultrastructural observations of the pupal cuticle and pharate adult cuticle of the uninjected control, GFP dsRNA-injected control and surviving Aacec B dsRNA-injected *Ae. aegypti* pupae. Edc: epidermal cell, aenv: pharate adult envelope, aepi: pharate adult epicuticle, aexo: pharate adult exocuticle. Note that at 12 hrs after injection, the pupal cuticle consisted of an envelope, an epicuticle, seven-laminated exocuticle and fifteen-laminated endocuticle; the pharate adult envelope began to be deposited on epidermal cells in uninjected control and GFP dsRNA-injected control mosquitoes, whilst the pupal cuticle consisted of an envelope, an epicuticle, seven-laminated exocuticle and twelve-laminated endocuticle and deposition of the pharate adult cuticle envelope was not observed in surviving Aacec B dsRNA-injected pupae. At 24 hrs after larval-pupal ecdysis, fourteen pupal endocuticlar lamellae remained; the envelope of the pharate adult cuticle was completely deposited in the uninjected control and GFP dsRNA-injected control mosquitoes, whilst eleven pupal endocuticlar lamellae remained; the pharate adult envelope was amorphous with numerous irregular granule-like particles in surviving Aacec B dsRNA-injected mosquitoes. At 36 hrs after injection, thirteen pupal endocuticlar lamellae remained; a 4-laminated exocuticle present in uninjected control and GFP dsRNA-injected control pupae and at the same time the chitin microfibrils were arranged in a helicoidal pattern. This contrasted with ten pupal endocuticlar lamellae remained; the only 1-laminated exocuticle present in the surviving Aacec B dsRNA-injected pupae, where the helicoidal pattern of chitin microfibrils was also disorganized. The boxed figures are higher magnifications of the pharate adult exocuticle showing the helicoidal pattern of the chitin microfibrils. Uncropped images are shown in Supplementary Figure S2.

Cuticle formation is a major event during pupal morphogenesis and therefore we investigated by electron microscopy if there were any ultrastructural changes in the pupal cuticle of the Aacec B-knockdown *Ae. aegypti*. The findings presented above indicate that knockdown of Aacec B results in high mortality rates at 120 hrs after Aacec B dsRNA injection. Therefore, at each time-point after Aacec B dsRNA injection, five survived pupae at least were randomly selected for ultrastructural study. As shown in Fig. 3, at 12 hrs after larval-pupal ecdysis, the pupal cuticle consisted of an envelope, an epicuticle, seven-laminated exocuticle and fifteen-laminated endocuticle; at this point in time the envelope of the pharate adult cuticle began to be deposited on the apical membrane of the epidermal cells. Such deposition of pharate adult cuticle envelope was not observed and the pupal cuticle consisted of an envelope, an epicuticle, seven-laminated exocuticle and twelve-laminated endocuticle in Aacec B dsRNA-injected pupae. At 24 hrs after larval-pupal ecdysis, fourteen endocuticlar lamellae remained; the envelope of pharate adult cuticle was completely deposited on the apical membrane of epidermal cells in the uninjected control and GFP dsRNA-injected control mosquitoes; this contrasted with the situation in the Aacec B dsRNA-injected pupae where eleven pupal endocuticlar lamellae remained; the pharate adult envelope was amorphous and contained numerous irregular, granule-like particles. At 36 hrs after larval-pupal ecdysis, thirteen pupal endocuticlar lamellae remained; the pharate adult cuticle consisted of an envelope, an epicuticle and one four-laminated exocuticle in the uninjected control and GFP dsRNA-injected control mosquitoes; furthermore, the chitin microfibrils in the fully formed exocuticle lamellae were arranged in an electron-dense helicoidal pattern within the chitin-protein matrix. However, in Aacec B dsRNA-injected pupae only ten pupal endocuticlar lamellae remained and only an envelope, an epicuticle and one-laminated exocuticle were deposited to form the pharate adult cuticle. Furthermore, the helicoidal pattern of the chitin microfibrils was disorganized and numerous irregular electron-lucent spaces were present in the chitin-protein matrix.

### Proteomic and expression analysis reveals that Aacec B knockdown reduces the expression levels of *Ae. aegypti* prophenoloxidases in pupae

**Figure 4:**
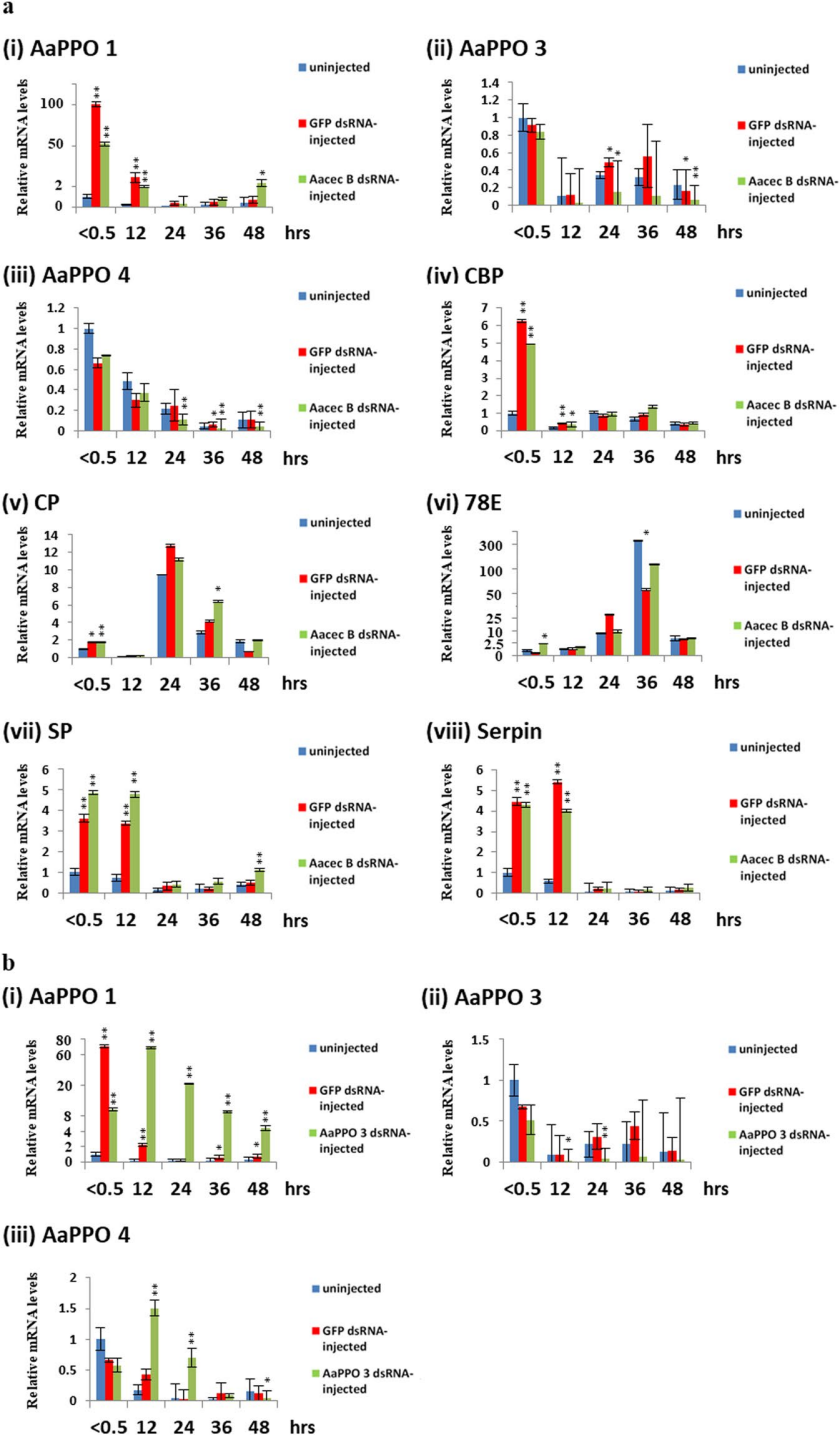
RT-qPCR analysis of the expression profiles of the proteins relates to cuticle formation. P_< 0.5_: <0.5 hr after injection; P_12_–P_48_: 12–48 hrs after pupation. The relative expression levels are expressed as means ± SD (n = 3), with uninjected pupae at <0.5 hr after injection as the calibrator. Asterisks indicate significant differences (*p < 0.005; **p < 0.001) from the relative mRNA levels of uninjected control at each time-point. (**a**) The expression profiles of eight proteins related to cuticle formation in uninjected control, GFP dsRNA-injected control and Aacec B dsRNA-injected pupae. AaPPO 1: *Ae. aegypti* prophenoloxidase 1; AaPPO 3: *Ae. aegypti* prophenoloxidase 3; AaPPO 4: *Ae. aegypti* prophenoloxidase 4; CBP: chitin binding protein; CP: cuticle protein; 78E: pupal cuticle protein 78E; SP: serine protease; Serpin: serine protease inhibitor. (**b**) RT-qPCR analysis of AaPPO 1, 3 and 4 expression in uninjected control, GFP dsRNA-injected control and AaPPO 3 dsRNA-injected pupae.

The above results indicated that cuticle formation was impaired in the Aacec B dsRNA-injected pupae. Subsequently, a proteomic analysis was carried out in order to identify changes in protein levels within the Aacec B knockdown pupae. Compared with those in the uninjected control, the levels of 144 proteins were found to be changed by more than 2-fold in the Aacec B dsRNA-injected pupae at 48 hrs after larval-pupal ecdysis, with 84 proteins being down-regulated and 60 proteins being up-regulated (Supplementary Table S1). Among these 144 proteins, 13 proteins were identified as being related to insect cuticle formation (Supplementary Table S2). Furthermore, reverse transcription-quantitative PCR (RT-qPCR) analysis of the Aacec B-knockdown *Ae. aegypti* showed that, compared with the uninjected mosquitoes, knockdown of Aacec B significantly reduced the expression levels of AaPPO 3 and AaPPO 4. Specifically, there was only a trace amount of AaPPO 3 mRNA detectable at any time point after larval-pupal ecdysis in the Aacec B-knockdown pupae (Fig. 4a). By way of contrast, knockdown of Aacec B was able to significantly induce the expression levels of two proteins (AaPPO 1, and serine protease (SP) (QIHRH0)) at 48 hrs after larval-pupal ecdysis. Furthermore, cuticle protein (CP) (Q16VF4) also showed increased expression at 36 hrs after larval-pupal ecdysis. Finally, pupal cuticle protein 78E (78E) (Q16TU0)) showed increased expression at 0.5 hr after larval-pupal ecdysis (Fig. 4a).

### Knockdown of AaPPO 3 in pupae results in high pupal mortality and impairment of cuticle formation

**Figure 5:**
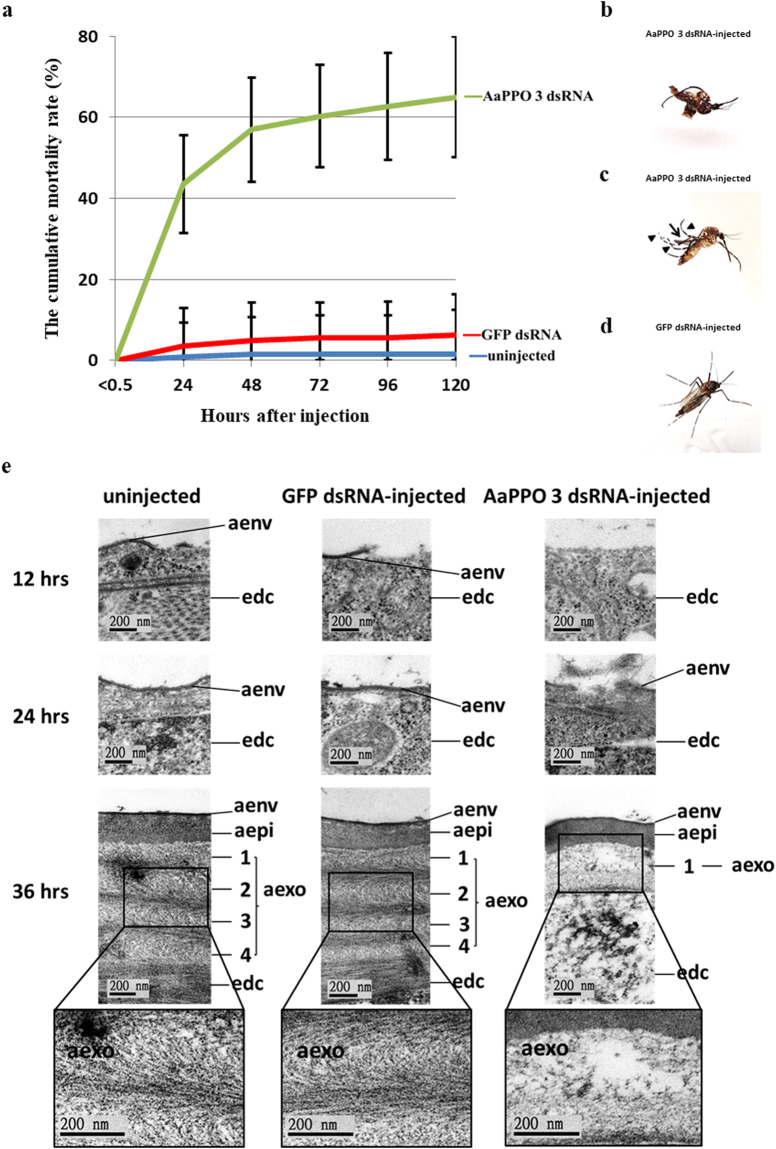
Knockdown of AaPPO 3 in *Ae. aegypti* pupae leads to high pupal mortality and the emergence of deformed adult. (**a**) The cumulative mortality rates (%) of uninjected control (blue line), GFP dsRNA-injected control (red line), AaPPO 3 dsRNA-injected (green line) *Ae. aegypti* pupae at various times after injection. P_< 0.5_: <0.5 hr after injection. 24–120: 24–120 hrs after larval-pupal ecdysis. Each group consisted of 20 pupae in one experiment and 10 replicates were conducted. The range bars indicate the standard deviations of means. (**b**) An adult mosquito unable to detach from exuvia of an AaPPO 3 dsRNA-injected pupa. (**c**) An adult with deformed wings and legs. Arrows (↑) indicate curved wings and arrow heads (▲) indicate curved legs. (**d**) An injection control mosquito. (**e**) Ultrastructural observations of the pharate adult cuticle in the uninjected control, GFP dsRNA-injected control and AaPPO 3 dsRNA-injected *Ae. aegypti* pupae. Edc: epidermal cell, aenv: pharate adult envelope, aepi: pharate adult epicuticle, aexo: pharate adult exocuticle. At 12 hrs after injection, the pharate adult envelope began to be deposited on the epidermal cells in uninjected control and GFP dsRNA-injected control mosquitoes, whilst the deposition of the pharate adult cuticle envelope was not observed in the AaPPO 3 dsRNA-injected pupae. At 24 hrs after larval-pupal ecdysis, the envelope of the pharate adult cuticle was deposited in the uninjected control and GFP dsRNA-injected control mosquitoes, whilst the pharate adult envelope was amorphous, containing numerous irregular granule-like particles, in the AaPPO 3 dsRNA-injected mosquitoes. At 36 hrs after injection, 4-laminated exocuticle was present in uninjected control and GFP dsRNA-injected control pupae, with the chitin microfibrils being arranged in a helicoidal pattern. By way of contrast, only an 1-laminated exocuticle was present in the AaPPO 3 dsRNA-injected pupae with the helicoidal pattern of the chitin microfibrils being disorganized. The boxed figures are higher magnifications of the pharate adult exocuticle showing the helicoidal pattern of chitin microfibrils. Uncropped images are shown in Supplementary Figure S3.

Tsao *et al*. (2010) demonstrated that a mosquito prophenoloxidase, *Armigeres subalbatus* prophenoloxidase III (As-pro-PO III), is required for cuticle formation in the pupae of *Ar. subalbatus* ^18^. They also suggested that AaPPO 3 is a homologous to As-pro-PO III^19^. In this context, we investigated the role of AaPPO 3 in cuticle formation by injecting AaPPO 3 dsRNA (745 bp, position from −56 to +689 bp) into pupae immediately after larval-pupal ecdysis. When compared with the uninjected control and GFP dsRNA-injected control mosquitoes, the transcription level of AaPPO 3 in the AaPPO 3 dsRNA-injected pupae was significantly reduced. However, it should be noted that injection of AaPPO 3 dsRNA significantly induced the transcriptional levels of AaPPO 1 at all time-points after larval-pupal ecdysis and also increased the transcriptional levels of AaPPO 4 at 12 and 24 hrs after larval-pupal ecdysis (Fig. 4b). These findings indicate that AaPPO 3 dsRNA is able to specifically knockdown the expression level of AaPPO 3 in pupae, but it is also able to induced the expression of AaPPO 1 and AaPPO 4. It was found that injection of AaPPO 3 dsRNA resulted in high pupal mortality and the emergence of deformed adults. As shown in Fig. 5a, at 120 hrs after AaPPO 3 dsRNA injection, the cumulative mortality rates of the GFP dsRNA-injected control and uninjected control mosquitoes were 6.31 ± 6.19% and 1.5 ± 3.19%, respectively, whilst the cumulative mortality rate of AaPPO 3 dsRNA-injected insects was 64.99 ± 14.82%, including 8.08% of the AaPPO 3 dsRNA-injected pupae that emerged as adults, but were unable to detach from the pupal exuvia (Fig. 5b). Furthermore, 6.83% of the AaPPO 3 dsRNA-injected pupae emerged as deformed adults with curved legs or wings (Fig. 5c) and died shortly after emergence. Only 28.19% of the AaPPO 3 dsRNA-injected pupae successfully emerged as normal adults. An ultrastructural analysis revealed that knockdown of AaPPO 3 also resulted in an impairment of cuticle formation, with fewer pharate adult exocuticle lamellae being deposited and the electron-dense helicoidal pattern of chitin microfibrils being disorganized. This resulted in an irregular formation of numerous electron-lucent spaces being present, together with the loss of the chitin-protein matrix (Fig. 5e). These findings indicate that AaPPO 3 is required for the formation of the pharate adult cuticle in pupae.

### Injection of exogenous Aacec B peptide into pupae elevates the transcription levels of AaPPO 3

**Figure 6:**
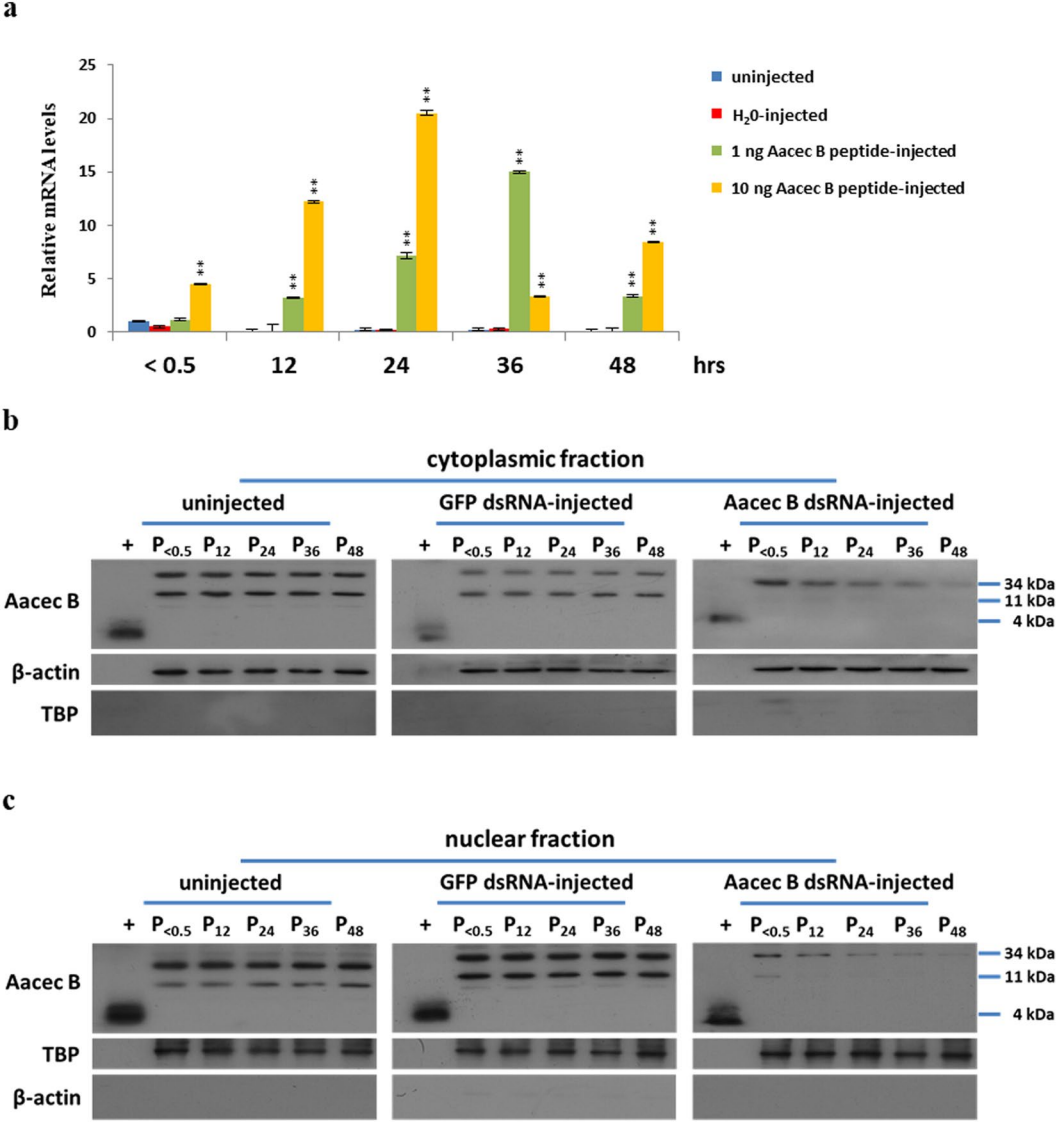
Exogenous Aacec B peptide is able to elevate the transcription levels of AaPPO 3, and Aacec B is able to be detected in the cytoplasm and nucleus of pupal cells. (**a**) The expression profile of AaPPO 3 in uninjected control, ddH_2_O-injected control, 1 ng or 10 ng of Aacec B peptide was injected into pupae. The relative expression levels are expressed as means ± SD (n = 3), with uninjected pupae at <0.5 hr after injection as the calibrator. Asterisks indicate significant differences (*p < 0.005; **p < 0.001) from the relative mRNA levels of uninjected control at each time-point. (**b,c**) Western blot analysis using antibodies specific for Aacec B: (**b**) cytoplasmic fraction and (**c**) nuclear fractions. β-actin and TATA-binding protein (TBP) were used as loading controls for the cytoplasmic and nuclear fractions, respectively. P_< 0.5_: <0.5 hr after injection; P_12_–P_48_: 12–48 hrs after pupation. +: synthetic Aacec B peptide. Uncropped images are shown in Supplementary Figure S4.

The above results show that knockdown of Aacec B and AaPPO 3 in *Ae. aegypti* pupae have similar effects on the pupae, namely high mortality (Figs. 2b and 5a), the emergence of deformed adults (Figs. 2d and 5c), and an impairment of cuticle formation (Figs. 3 and 5e); in both cases the transcription level of AaPPO 3 was significantly reduced (Fig. 4). In the light of these findings, we propose that Aacec B may act as a transcriptional factor (TF) and regulate the gene expression of AaPPO 3 in the pupae. To investigate this hypothesis, exogenous Aacec B peptide was injected into pupae immediately after larval-pupal ecdysis to investigate the protein’s effect on the expression of AaPPO 3. As shown in Fig. 6a, the expression levels of AaPPO 3 in pupae injected with 1 ng and 10 ng of Aacec B peptide resulted in a significant elevation of the expression of AaPPO 3 at each time point from 12 hrs to 48 hrs after injection.

### Aacec B is present in the cytoplasm and nucleus of pupal cells

One important feature of a TF is the fact that a TF is able to be translocated into nucleus, after which the TF binds to appropriate DNA-binding sites within its target genes^20^. As shown in Fig. 1c, we found that two bands, with molecular weights correspond to the trimer and nonamer of Aacec B, were presented in total protein extract of mosquito cells. Subsequently, Western blotting was able to detect the presence of Aacec B in the nuclei of pupal cells. As shown in Figs. 6b and 6c, when the uninjected control and GFP dsRNA-injected control pupae were examined, both of these bands were able to be detected in the cytoplasmic fraction and in the nuclear fraction, with the protein levels of the bands in cytoplasmic fraction with a molecular weight corresponding to that of the trimer of Aacec B being higher than those of the bands corresponding to the molecular weight of the nonamer of Aacec B (Fig. 6b). By way of contrast, in the nuclear fraction the protein level of the bands with a molecular weight corresponding to that of the nonamer of Aacec B were more abundant than that of the bands with molecular weight corresponding to that of the trimer of Aacec B (Fig. 6c). Importantly, in both the cytoplasmic and nuclear fractions of Aacec B dsRNA-injected pupae, the bands with a molecular weight corresponding to that of the nonamer of Aacec B gradually decreased when measured at 12, 24, 36 and 48 hrs after larval-pupal ecdysis. Furthermore, only a small amount of protein with a molecular weight corresponding to that of the trimer of Aacec B was able to be detected in the nuclear fractions of the Aacec B dsRNA-injected pupae at 0.5 hrs after larval-pupal ecdysis (Figs. 6b and 6c).

### Aacec B directly binds to a putative binding motif TTGG(A/C)A present in four DNA positions within the AaPPO 3 gene, namely +245 to +250 bp, +732 to +737 bp, +1307 to +1312 bp, and +1369 to +1374 bp

**Figure 7:**
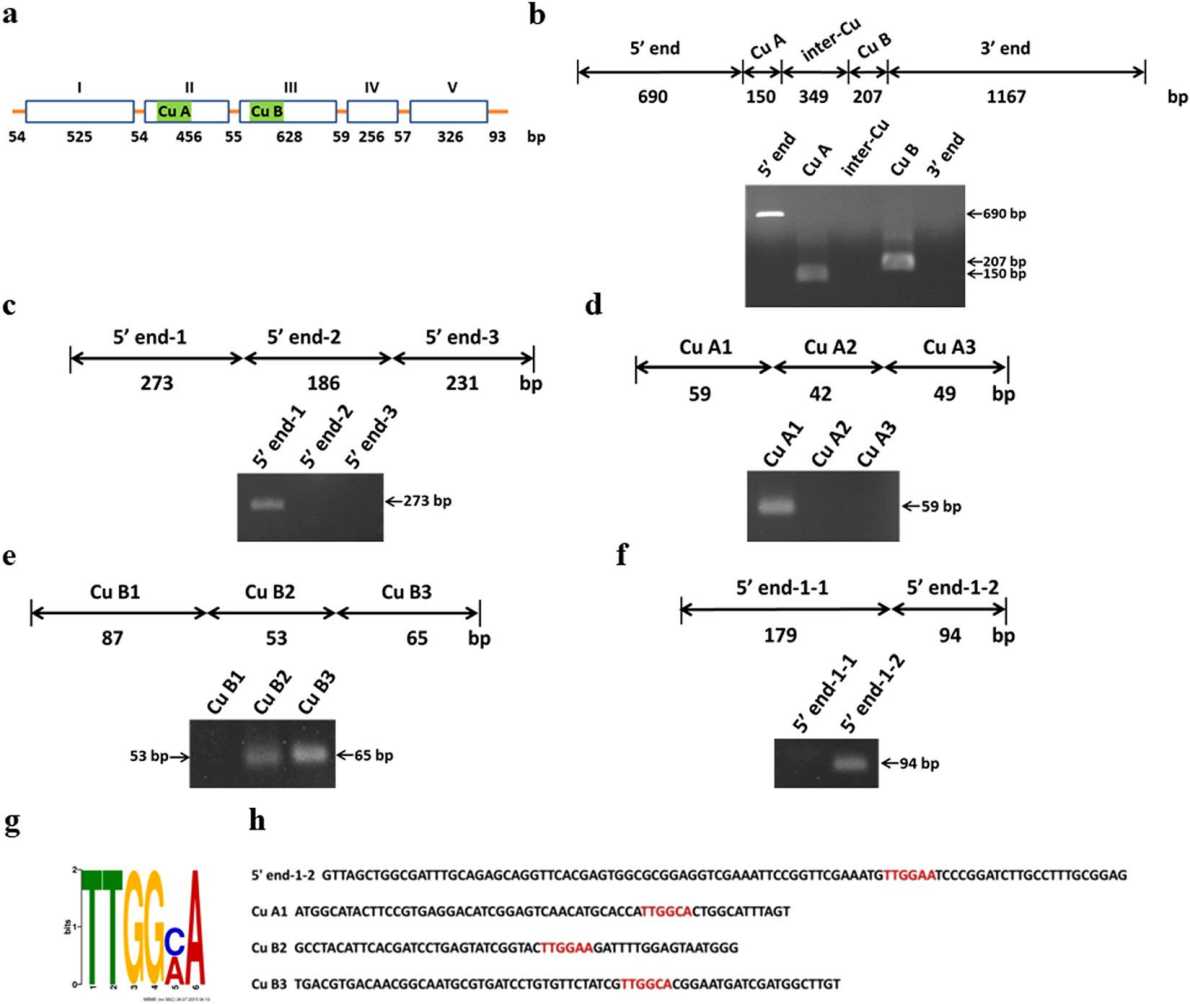
Aacec B peptide binds to the putative binding motif TTGG(A/C)A present in four DNA fragments of AaPPO 3. (**a**) A schematic representation of the AaPPO 3 gene. Open boxes with roman numerals indicate exons and the nucleotide length of introns and exons are indicated by numbers. The green boxes indicate the copper binding site Cu A and Cu B. (**b**–**f**) DNA pull-down assay showing Aacec B peptide binding to AaPPO 3 DNA. The upper figures show schematic representations of the AaPPO 3 DNA fragments. The nucleotide lengths are indicated by numbers. The lower figures are the PCR analyses of the AaPPO 3 DNA fragments that are able to bind to Aacec B-conjugated beads. The arrows point to the PCR amplifications of the AaPPO 3 DNA fragments binding to the Aacec B-conjugated beads. (**g**) DNA sequence logo representing the TTGG(A or C)A binding motif that was identified from the four AaPPO 3 DNA fragments (5′ end-1-2, Cu A1, Cu B2 and Cu B3) using the MEME suite. The height of each letter represents the relative frequency of occurrence of the nucleotide at each position. (**h**) The nucleotide sequences of the four AaPPO 3 DNA fragments (5′ end-1-2, Cu A1, Cu B2 and Cu B3) containing the TTGGAA and TTGGCA putative motifs (indicated in red). Uncropped images are shown in Supplementary Figure S5.

The above results have demonstrated that Aacec B is able to be detected in both the cytoplasm and nucleus of pupal cells (Figs. 6b and 6c). Next we used a DNA pull-down assay to determine whether the Aacec B peptide was able to bind AaPPO 3 DNA *in vitro*. The AaPPO 3 gene sequence (AAEL011763) was obtained from VectorBase (http://www.vectorbase.org). The AaPPO 3 gene consists of five exons (exon I, 525 bp; exon II, 456 bp; exon III, 628 bp; exon IV, 256 bp; exon V, 326 bp) separated by four short introns (intron I, 54 bp; intron II, 55 bp; intron III, 59 bp; intron IV, 57 bp). Two highly conserved copper-binding sites, Cu A and Cu B, are located in exon II and exon III, respectively (Fig. 7a). Five DNA fragments of AaPPO 3, namely (1) the untranslated region and 5′ end region (690 bp), (2) the Cu A region (150 bp), (3) the region between the two copper-binding sites (the inter-Cu region) (349 bp), (4) the Cu B region (207 bp), and (5) the 3′ end and a part of the poly(A) tail region (1167 bp), were cloned after PCR. Subsequently, the binding of the Aacec B peptide to these five AaPPO 3 DNA fragments was investigated by DNA pull-down assay. As showed in Fig. 7b, the Aacec B peptide was able to bind three of these DNA fragments, specifically the 5′ end, Cu A, and Cu B fragments. Next each of these three DNA fragments was further subdivided into three shorter fragments. Further DNA pull-down assays found that Aacec B bound to four of these smaller AaPPO 3 DNA fragments. These were the +1 to +273 bp region within the 5′ end-1 DNA fragment, the +691 to +749 bp region within the Cu A1 DNA fragment, the +1277 to +1329 bp region within the Cu B2 DNA fragment, and the +1330 to +1394 bp region within the Cu B3 DNA fragment (Fig. 7c–e). The 5′ end-1 DNA fragment was further divided into two shorter fragments and it was found that Aacec B was able to bind to the +180 to +273 bp region within the 5′ end-1-2 DNA fragment (Fig. 7f). The MEME suite (http://meme-suite.org/tools/meme) was next used to identify the most statistically significant common motifs within the four AaPPO 3 DNA fragments (5′ end-1-2, Cu A1, Cu B2 and Cu B3) that might be involved in the binding of Aacec B. One putative Aacec B binding motif, TTGG(A or C)A, was identified (Fig. 7g). The positions of the TTGG(A or C)A putative motif were at +245 to +250 bp region within the 5′ end-1-2 DNA fragment, at +732 to +737 bp within the Cu A1 DNA fragment, at +1307 to +1312 bp within the Cu B2 DNA fragment, and at +1369 to +1374 bp within the Cu B3 DNA fragment (Fig. 7h).

### Aacec B peptide binds *in vitro* to the TTGG(A/C)A motif present in AaPPO 3 DNA, and the TTGG(A/C)A motif is also present in the genes encoding AaPPO 1 and AaPPO 4

**Figure 8:**
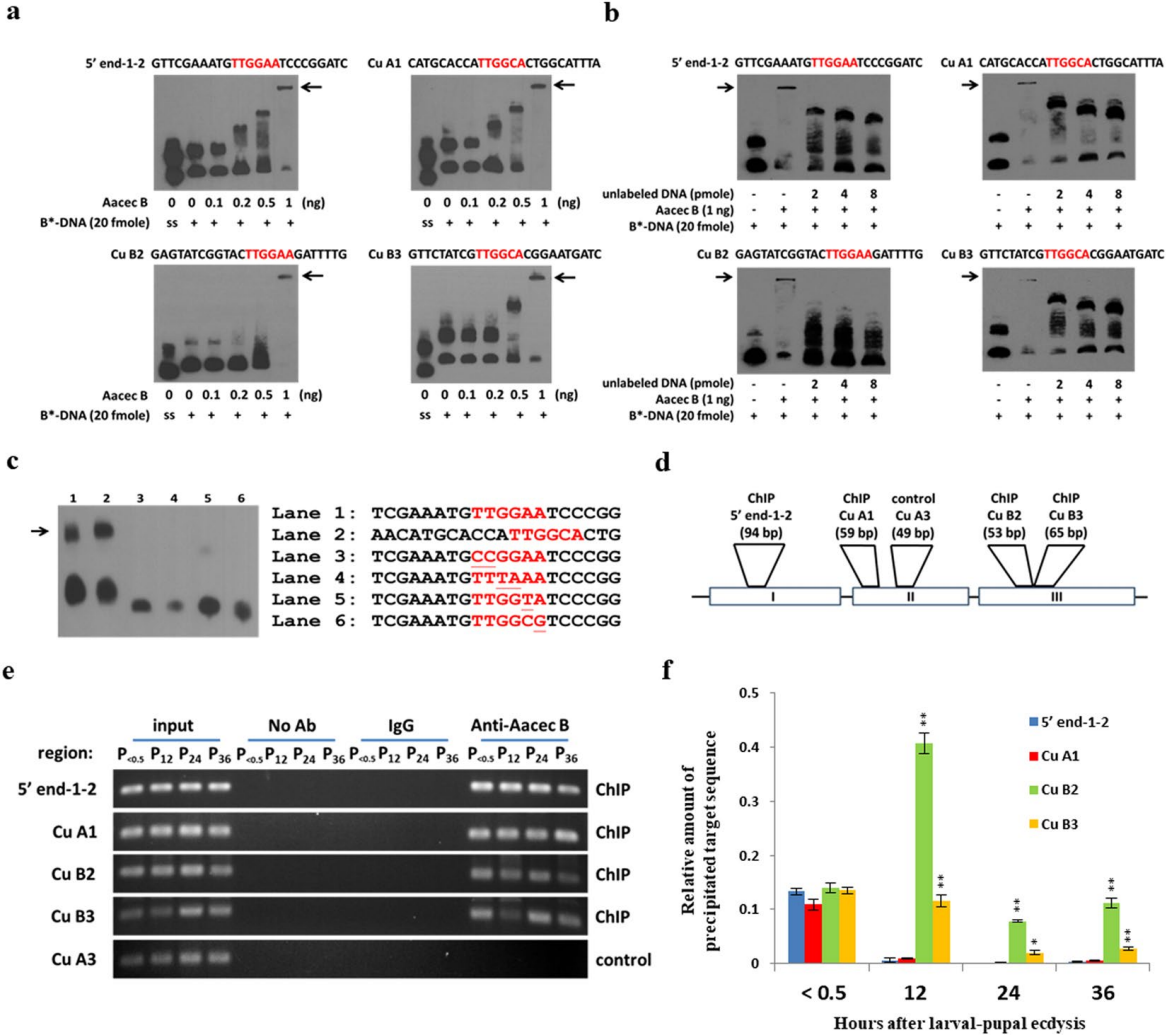
The results of EMSA analyses and ChIP assay reveal that Aacec B peptide binding to the TTGG(A or C)A putative binding motifs of the four AaPPO 3 DNA fragments. (**a–c**) EMSA analysis results. The TTGGAA and TTGGCA putative motifs are indicated in red. The arrows point to the position of shifted bands. B*-DNA: Biotin-labeled DNA. ss: biotin labeling-single stranded DNA. (**a**) EMSA analyses showed that a shifted band was detected when each of the four AaPPO 3 DNA fragments containing TTGG(A or C)A putative binding motif were incubated with 1 ng Aacec B peptide. (**b**) Competition assays showed that the shifted bands of the four AaPPO 3 DNA fragments containing the TTGG(A or C)A putative binding motifs are able to be competed with when increasing concentrations of unlabeled DNA fragment (2 to 8 pmole) is added. (**c**) EMSA analysis showing that the shifted bands of two AaPPO 3 DNA fragments containing either the TTGG(A or C)A putative binding motif (indicated in red) can be detected (lanes 1 and 2), but no shifted band was detected after incubation with Aacec B peptide when the DNA fragments used contained nucleotide replacements within the TTGG(A or C)A motifs (indicated by single underline) (lanes 3–6). (**d–f**) The ChIP assay results. (**d**) Schematic representation of the ChIP and control primers is shown. Open boxes with roman numerals indicate exons. (**e**) ChIP assay on the four AaPPO 3 DNA fragments containing the TTGG(A or C)A putative binding motif. Aacec B antibody was used for the ChIP assay. No antibody (no Ab), normal rabbit IgG and Cu A3 DNA fragment were used in this experiment as negative controls. Three independent experiments were done with the similar results. Results from one experiment are shown. (**f**) qPCR of ChIP assay (**e**). qChIP results are presented as the mean of three independent experiments (n = 3; Mean ± SD). Asterisks indicate significant differences (*p < 0.005; **p < 0.001) from the signals from the TTGGAA motif within the 5′ end-1-2 DNA fragments at each time-point. Uncropped images are shown in Supplementary Figures S6 and S8. In (**a**), (**b**) and (**c**), similar results are seen in the other two independent experiments (see also Supplementary Figure S6b and S6c).

Subsequently, the ability of the Aacec B peptide to bind the TTGG(A or C)A putative binding motifs was further validated using electrophoretic mobility-shift assays (EMSA). As shown in Fig. 8a, incubation of 1 ng Aacec B peptide with the four biotin-labeled AaPPO 3 DNA fragments containing the putative TTGG(A or C)A motifs resulted in a single retarded band. Subsequently, a competition assay was used to examine the specificity of binding of the TTGG(A or C)A motifs to the Aacec B peptide. As shown in Fig. 8b, unlabeled DNA fragments containing TTGG(A or C)A motif were able to successfully compete with the binding of Aacec B to the four biotin-labeled AaPPO 3 DNA fragments.

Next four AaPPO 3 DNA fragments containing the binding motifs were modified by replacing TT with CC, by replacing GG with TA, by replacing (A or C)A with TA and by replacing (A or C)A with CG and then the binding affinities of the Aacec B peptide to the modified motifs was investigated in detail. As shown in Fig. 8c, incubation of Aacec B peptide with the DNA fragments containing TTGGAA and TTGGCA resulted in a single shifted band. However, no shifted band was observed after incubation of the Aacec B peptide with the DNA fragments containing the nucleotide replacements within the TTGG(A or C)A motif. These results indicate that the Aacec B peptide is able to specifically bind to the TTGG(A or C)A motifs. When further MEME suite analysis was carried out, it revealed that three TTGG(A/C)A motifs were also present in the gene encoding AaPPO 1 and in the gene encoding AaPPO 4. These motifs were present at +206 to +251 bp within the AaPPO 1-5′ end DNA fragment, at +700 to +746 bp within the AaPPO 1-Cu A DNA fragment, at +1289 to +1334 bp within the AaPPO 1-Cu B DNA fragment, at +688 to +733 bp within the AaPPO 4-Cu A DNA fragment, at +8334 to +8379 bp within the AaPPO 4-Cu B DNA fragment, and at +8396 to +8441 bp within the AaPPO 4-Cu B DNA fragment (Supplementary Figure S7).

### Aacec B peptide binds to the TTGG(A/C)A motif in AaPPO 3 DNA *in vivo* at 12 hrs after larval-pupal ecdysis

In addition, chromatin immunoprecipitation (ChIP) assays and quantitative PCR were used to confirm the direct binding of Aacec B peptide to the four AaPPO 3 DNA fragments containing TTGG(A or C)A motif. As shown in Fig. 8e, the four AaPPO 3 DNA fragments containing TTGG(A or C)A motif. Specifically, 5′ end-1-2, Cu A1, Cu B2 and Cu B3,were significantly enriched in the ChIP fractions compared with the no antibody, normal IgG and Cu A3 DNA fragment assay, which were used as negative control groups. Subsequently, quantitative PCR was used to analyze Aacec B interactions with the four AaPPO 3 DNA fragments containing TTGG(A or C)A motif. As shown in Fig. 8f, the signals from the three TTGG(A or C)A motif within the 5′ end-1-2 DNA, Cu A1 and Cu B3 fragments were detected immediately after larval-pupal ecdysis and had decreased at 12 hrs after larval-pupal ecdysis. However, the signal due to the TTGGAA motif within the Cu B2 DNA fragment was significantly increased compared with that of the other three AaPPO 3 DNA fragments containing TTGG(A or C)A motif at 12 hrs after larval-pupal ecdysis and then had decreased at 24 hrs and 36 hrs after larval-pupal ecdysis.

## Discussion

Up to the present more than 40 insect cecropins have been identified and described in the orders of Coleoptera^21^, Diptera^11–17,22–25^ and Lepidoptera^26–38^. Most cecropins are inducible and exhibit broad-spectrum antimicrobial activity against both Gram (+) and Gram (−) bacteria; they do this by forming ionic pores that are able to destroy the ionic balance of the invading bacteria^38,39^. In addition, cecropins have also been shown to be able to impede the development of oocysts of *Plasmodium* in *Anopheles* mosquitoes^40^ and to reduce the motility of *Brugia pahangi* microfilariae in *Ae. aegypti* ^41^. In this study, we have demonstrated that Aacec B plays a significant role in the pharate adult cuticle formation of *Ae. aegypti* pupae, a novel function for an AMP. We have also found that knockdown of Aacec B in pupae results in high mortality (Fig. 2b), the emergence of deformed adults (Fig. 2d) and an impairment of pharate adult cuticle formation. The last involves the deposition of fewer lamellae within the pharate adult exocuticle and disorganization of the helicoidal pattern of chitin microfibrils (Fig. 3). Furthermore, we found that pupal mortality was significantly reduced and no deformed adults emerged after pupae were simultaneously injected with Aacec B dsRNA and Aacec B peptide, which indicates that Aacec B is required for pharate adult cuticle formation (Fig. 2b). We also found that the transcription of AaPPO 3 and 4 were significantly decreased in the Aacec B-knockdown pupae (Fig. 4a). Phenoloxidases (PO) are oxidases that contain multiple atoms of copper and has long been suggested to play important roles in various physiological functions of insects, including cuticular sclerotization, cuticle formation, egg tanning, wound healing, the melanotic encapsulation of parasites^18,42–49^, etc. POs are present in mosquitoes as an inactive form, namely PPOs. Multiple PPOs are known to be expressed in mosquitoes with the genomes of *Ae. aegypti*, *An. gambiae* and *Culex quinquefasciatus* containing ten, nine and nine genes, respectively, and these; are thought to encode these prophenoloxidases^42,43^. Although the genome of *Ar. subalbatus* has not been sequenced, five As-pro-POs have been identified in this species^44^. Different PPOs may have different functions, for example in *Ar. subalbatus*, the functions of the As-pro-PO I, II and III were found to be involved in filarial parasite melanization, blood feeding and cuticle formation, respectively^18,45,46^, while As-pro-PO V was required for both melanization immune responses and egg chorion melanization^44^. Tsao *et al*. (2009) also suggested that AaPPO 3 is homologous to As-pro-PO III^19^. In this study, we have demonstrated that AaPPO 3 also plays a significant role in pharate adult cuticle formation in the pupae of *Ae. aegypti*. Our research shows that knockdown of AaPPO 3 in *Ae. aegypti* pupae and results in similar effects to that of As-pro-PO III-knockdown in *Ar. subalbatus* pupae, namely a high mortality, the emergence of deformed adults and an impairment of cuticle formation.

Further investigation showed that Aacec B would seem to act as a TF and affect the transcription of AaPPO 3. Several areas of our results support this hypothesis. Firstly, injection of exogenous Aacec B peptide into pupae was found to significantly up-regulate the transcription of AaPPO 3 (Fig. 6a). Secondly, Aacec B peptide is able to be detected in the cell nuclei of pupae (Figs. 6b and 6c). Thirdly, Aacec B is able to bind *in vitro* to multiple putative binding motifs, namely TTGG(A/C)A, within the AaPPO 3 DNA sequence (Figs. 7 and 8a). The specificity of the binding of the Aacec B peptide to the putative motif TTGG(A/C)A was confirmed by competition assay (Fig. 8b) and nucleotide replacement analysis (Fig. 8c). Finally, the ChIP analyses confirmed that Aacec B did bind to the four putative motif TTGG(A/C)A in AaPPO 3 DNA *in vivo* (Fig. 8e). Quantitatively, the signal from the TTGGAA motif within the Cu B2 DNA fragment was higher than the other three putative motif TTGG(A/C)A in AaPPO 3 DNA at 12 hrs after larval-pupal ecdysis (Fig. 8f). The nucleotide sequence of the putative binding motif TTGG(A/C)A is almost identical to that of the consensus motif TTGGCA of the mammalian NFI family transcription factors. The NFI family has been suggested to be essential for mammalian development^50,51^. Based on the above it is reasonable to assume that the TTGG(A/C)A sequences with AaPPO 3 are functional and are involved in controlling the expression of this development-related gene.

In conclusion, our results support the idea that Aacec B plays a crucial role in pharate adult cuticle formation and does this via the regulation of AaPPO 3 gene expression in pupae. In addition to AaPPO 3, three TTGG(A/C)A putative binding motifs were also found to be present in the coding sequences of AaPPO 1 and AaPPO 4 (Supplementary Figure S7) and it is interesting that we have found that knockdown of Aacec B also decreased the transcription levels in pupae of AaPPO 4 (Fig. 4a). However, knockdown of AaPPO 3 by AaPPO 3 dsRNA also induced the expressions of AaPPO 1 and AaPPO 4 (Fig. 4b). This induction might have been caused by the injection of AaPPO 3 dsRNA into the mosquitoes causing tissue damage, AaPPO 3 dsRNA blocked the transcription of AaPPO 3 that is initiated via the binding of Aacec B. Alternatively, Aacec B might bind to the TTGG(A/C)A putative binding motifs of AaPPO 4 to elevate transcription of AaPPO 4 and, furthermore, the TTGG(A/C)A putative binding motifs is also present in the gene encoding AaPPO 1. Further studies are needed to verify whether Aacec B also acts as a TF for AaPPO 1 and 4.

Durell *et al*. (1992) used an atomic-scale computer model to demonstrate that six cecropin dimers are required to produce a reasonable large pore that ought to be capable of conducting ions^52^. In this study, we found that two bands with molecular weights corresponding to the trimer of Aacec B and to the nonamer of Aacec B were detected by anti-Aacec B antibody in total protein extract, in the cytoplasm of pupal cells and in the nuclei of pupal cells. Although it is strange to find bands corresponding to polymeric forms of the protein in gels run under reducing conditions, the fact that those bands disappear in Aacec B dsRNA-treated animals reinforces the idea that those bands genuinely correspond to Aacec B. However, the nature of these two bands needs to be investigated further in the future. It would be very interesting to know whether it is the trimeric or the nonameric form of the Aacec B peptide that acts as a TF and controls the expression of AaPPO 3.

## Materials and Methods

### Mosquito rearing

The *Ae. aegypti* mosquitoes reared at National Taiwan University were collected by Dr. Tsai at Kaoshiung City, Taiwan, in 1998. This strain of mosquito has been maintained in our insectary since that time. Mosquitoes are reared at 28 ± 1 °C and 70–80% RH on a 12-h photoperiod under standard laboratory conditions. Adult mosquitoes are provided a 10% sugar solution, and females are blood fed on anesthetized mice bi-weekly. Larvae are fed a mixture of goose liver powder and yeast powder (1:1).

### Preparation of killed bacteria and injection of the bacteria into the mosquitoes

Injection of bacteria into mosquitoes was carried out as previously described^11^. Gram (−) *Escherichia coli* BL21 and Gram (+) *Staphylococcus aureus* CCRC 15211 cultures were grown overnight in LB broth at 37 °C, and then heat-killed bacteria were obtained by holding the culture at 100 °C for 10 min. The preparations containing killed bacteria were then stored at −70 °C until use. About 1 × 10^8^ CFU/ml heat-killed bacteria were injected into the thorax of pupae and adult female mosquitoes using an injector linked to a glass capillary needle.

### RNA extraction

The whole mosquito bodies were homogenized in 1 ml RNAzol^TM^ B buffer (Biotecx Laboratories Inc, USA) and mixed with 200 μl chloroform; they were then placed on ice for 20 min, which was followed by centrifuged for 20 min at 12,000 g at 4 °C. The aqueous layer was transferred to a new tube and mixed with an equal volume of isopropanol on ice for 15 min and centrifuged for 20 min at 12,000 g at 4 °C. The pellet was washed with 1 ml of 75% ethanol, dried for 2 min at 65 °C, and finally dissolved in DEPC-treated water. The RNA concentration was measured using a NanoDROP^TM^ ND-1000 spectrophotometer (Thermo Scientific, USA), and the final RNA samples were stored at −80 °C.

### Reverse transcription-quantitative PCR (RT-qPCR) analysis

Gene expression analysis was performed by two steps RT-qPCR. A RevertAid^TM^ First Strand cDNA Synthesis Kit (Fermentas, USA) was used to prepare cDNA from the RNA samples according to the manufacturer’s protocol. The sequences of the ten Aacecs and the eight proteins related to cuticle formation expressed by *Ae. aegypti* were obtained from VectorBase (http://www.vectorbase.org). The specific primer pairs used for the RT-qPCR are shown in Supplementary Tables S3–S4. Ribosomal protein S7 was amplified with a pair of specific primers as the internal control. The sequences of the PCR products amplified by the specific primer pairs were confirmed by sequencing. The quantitative PCR was performed using a StepOnePlus™ System (Applied Biosystems, USA) in 96-well plates containing 10 μl of Luminaris Color HiGreen qPCR Master Mix (Thermo Scientific, USA), 2.5 μL of 1.6 μM of each gene-specific primer, and 5 μL of diluted cDNA in a final volume of 20 μL. The standard amplification parameters consisted of an initial activation step at 95 °C for 3 min; and then 40 cycles at 95 °C for 15 s for denaturation; 54 °C for 30 s for primer annealing. The data obtained from the RT-qPCR were analyzed by one-tailed t-test, with a value of *p* < 0.005 being used to evaluate significant differences. Each sample was analyzed in triplicate.

### Double-stranded RNA synthesis and injection

The construction and preparation of dsRNA were carried out as previously described^19^. The primer sequences and PCR condition are listed in Supplementary Table S3. In brief, the double-stranded RNA (dsRNA) of Aacec B was synthesized using either a T3 or T7 Megascript transcription kits (Ambion, USA). About 1 μg of dsRNA was dissolved in 0.5 μl DEPC-treated water and this was used to inject the thorax of pupae using an injector linked to a glass capillary needle. One group of control mosquitoes were injected with GFP dsRNA. Pupae were injected within 0.5 hr after larval-pupal ecdysis, and then total RNA from whole bodies was collected at various different time intervals after injection. The effects of gene silencing on the target genes were detected by RT-qPCR. The mortality rates of the dsRNA-injected and uninjected control mosquitoes were compared using two-tailed t-tests (*p* < 0.01).

### Total protein extraction and cytoplasmic and nuclear protein extraction

Total mosquito protein extracts were obtained by gently homogenizing whole mosquitoes in RIPA buffer (Bioman, Taiwan) containing a protease inhibitor cocktail (Calbiochem, USA) (1:1,000) that had been placed on ice for 30 min. Insoluble material was removed by centrifugation at 12,000 g at 4 °C and the supernatant was used for protein quantification. The cytoplasmic and nuclear protein extraction was performed using NE-PER nuclear and cytoplasmic Extraction Reagents (Thermo scientific, USA) by following the manufacturer’s protocol.

### SDS-polyacrylamide gel electrophoresis (SDS-PAGE) and Western blotting

Western blotting was carried out as previously described^53^. All samples were denatured by heating to 95 °C for 10 min and then resolved by 10% SDS-PAGE under reducing condition at 70 volt and 110 volt for the stacking gel and running gel, respectively. The proteins were then transferring to a polyvinylidene difluoride (PVDF) membrane (Millipore, USA). Anti-β-actin antibody (Santa Cruz Biotechnology, USA) and secondary anti-rabbit immunoglobulin (IgG)-horseradish peroxidase (HRP) (KPL, USA) were used for Western blot analysis. BLAST was used to search the most variable regions of the peptide sequence across the ten Aacecs. Next we selected the peptide sequence region near the C-terminal end (amino acid sequence N-FNAAQKGLPVAAGIKGLGR-C) and commissioned Yao-Hong Biotechnology Inc. (Taiwan) to synthesize the target peptide for use as an antigenic peptide. They subsequently generated the rabbit polyclonal anti-Aacec B antibody.

### Protein synthesis

The synthetic peptides used in this study were synthesized by Genemed Synthesis Inc., USA. The amino acid sequence of Aacec B peptide used in this study was N-GRLKKLGKKIERAGKRVFNAAQKGLPVAAGIKGLGR-C.

### In-gel digestion, protein identification using liquid chromatography-tandem mass spectrometry (LC-MS/MS) and mass spectrometric data analysis

The procedures were carried out as previously described^54^. In total, 50 μg of total protein were separated by 10% SDS-PAGE, which was followed by in-gel digestion. The peptide mixtures produced from the total mosquito protein extracts were analyzed by injection in triplicate into a nanoflow Ultra high-performance LC (nUPLC) system (Agilent Technologies, USA) coupled to a LTQ-Orbitrap Discovery™ hybrid mass spectrometer with a nanoelectrospray ionization source (Thermo Electron,USA). Additional filtering was carried out to allow protein identification and this involved having at least two unique peptides that matched with the Xcorr score for each peptide (γ 2.5). Label-free quantitative analysis using MS spectra counting involved the use of an in-house tool within the Microsoft VBA environment. Tandem MS spectral counts were normalized against the sum of the spectral counts per biological sample in quantitative analyses. *Ae. aegypti* protein sequences were accessed via the NCBI’s database (https://www.ncbi.nlm.nih.gov/) and the VectorBase website (http://www.vectorbase.org) using the dataset identifier. Details are provided in the supplementary information.

### Transmission electron microscopy

Sample preparation for electron microscopy was the same as previously described^18^. In brief, at least five surviving pupae were randomly selected at 12, 24 and 36 h after injection and dissected in cold PBS. The third and fourth abdominal fragments were excised and fixed with 3% glutaraldehyde (Electron Microscopy Science, USA) in cacodylate buffer (0.1 M, pH 7.4) (Merck, Germany), for 3 h at 4 °C and then post-fixed in 1% osmium tetroxide in cacodylate buffer (0.1 M, pH 7.4) for 6 h at 4 °C. After washing three times in cold PBS for 10 min each, the fixed specimens were dehydrated in increasing ethanol concentrations and then embedded in Epon 812 resin (Electron Microscopy Science, USA). The blocks were sectioned using an ultramicrotome (Ultracut; Leica Microsystems, Germany). The ultrathin sections were placed on formvar-coated copper grids, post-stained with 2% uranyl acetate (Merck, USA) and 1% lead citrate (Electron Microscopy Science, USA), and then visualized/photographed using a 100-kV transmission electron microscope (JEOL 2000EX II) (Japanese Electron Optic Laboratory, Japan).

### DNA pull-down assay

The DNA pull-down assay was carried out as described by Jutras *et al*.^55^ with some modifications. In this study, we used protein as bait to pull down the relevant DNA. Synthetic Aacec B peptide was covalently crosslinked with TANBead^®^ USPIO-101 (Taiwan Advanced Nanotech Inc., Taiwan) using 1-Ethyl-3-[3-dimethylaminopropyl] carbodiimide hydrochloride (EDC) (Sigma-Aldrich, USA) as the coupling buffer (100 mM MES (2-[N-morpholino] ethane sulfonic acid, 150 mM NaCl, pH 6.0) and then stored at 4 °C until use. The Aacec B peptide-conjugated magnetic beads were incubated with each AaPPO 3 DNA fragment for 30 min at 25 °C under gentle rotation. The Bead-Aacec B peptide-DNA fragment complexes were then collected using a magnet and the supernatant removed. Each sample was washed three times with 1X cold PBS plus 0.1% Tween-20, followed by two times with 1X cold PBS. Each Bead-Aacec B peptide-DNA fragment complex fraction was then collected again with a magnet and suspended in 50 μL distilled water for subsequent PCR analysis. The specific primer pairs used to detect the AaPPO 3 DNA fragments are shown in Supplementary Table S5.

### DNA binding motif analysis

The putative binding motifs of Aacec B-DNA binding sequence were identified using the MEME suite 4.10.2 (http://meme-suite.org/tools/meme) as previously described^56^. The parameters were as follows: one occurrence per sequence on given strand of DNA only and a width of 6 to 50 nucleotides.

### Electrophoretic mobility-shift assay (EMSA)

These assays were carried out as previously described^19^. Biotin end-labeled double-stranded oligonucleotide containing the TTGG(A/C)A consensus sequence was generated by annealing two single-stranded oligonucleotides. The sequences of the single-stranded oligonucleotides used for the EMSA are shown in Supplementary Tables S5–S6. For the competition experiments, the reactions were carried out by adding unlabeled double-stranded oligonucleotide containing the TTGG(A/C)A consensus sequence. All reaction mixtures were separated by electrophoresis on a 6% native polyacrylamide gel. The proteins were subsequently transferred to a nylon membrane and evaluation was carried out using a LightShift^®^ Chemiluminescent EMSA Kit (Pierce, USA).

### Standard and quantitative chromatin immunoprecipitation (ChIP and qChIP)

Standard ChIP analysis was carried out as previously described^57,58^. Briefly, *Ae. aegypti* pupae were homogenized in PBS on ice, which was followed by incubated with 1% formaldehyde to cross-link the DNA-protein complexes at 37 °C for 10 min and the cross-linking reaction was stopped by the addition of glycine. The ChIP assay was performed using the Pierce™ Magnetic ChIP Kit (Thermo Scientific, USA) according to the manufacturer’s instructions. The qChIP assay was performed using three biological replicates, and the amount of precipitated DNA was calculated as a percentage of the input sample. The primers were used in ChIP and qChIP assays are listed in Supplementary Tables S5–S6.

## Electronic supplementary material

Supplementary Information
