# Supplementary material for: Involvement of cecropin B in the formation of the Aedes aegypti mosquito cuticle
Source: Sci Rep. 2017 Nov 27;7:16395. doi: 10.1038/s41598-017-16625-6 (PMC5703890; doi:10.1038/s41598-017-16625-6)
Supplement: Supplementary file 1 — Supplementary Information [file 41598_2017_16625_MOESM1_ESM.pdf]

Supplementary Information

Involvement of cecropin B in the formation of the *Aedes aegypti* mosquito cuticle

Wei-Ting Liu[1], Wu-Chun Tu[2], Chao-Hsiung Lin[3], Ueng-Cheng Yang[4] & Cheng-Chen Chen[1]

[1]Institute of Microbiology and Immunology, National Yang-Ming University, Taipei, Taiwan, ROC.

[2]Department of Entomology, National Chung Hsing University, Taichung, Taiwan, ROC.

[3]Department of Life Sciences and Institute of Genome Sciences, National Yang-Ming University, Taipei, Taiwan, ROC.

[4]Institute of Biomedical Informatics, National Yang-Ming University, National Yang-Ming University, Taipei, Taiwan, ROC.

Correspondence and requests for materials should be addressed to

Dr. Cheng-Chen Chen

Institute of Microbiology and Immunology

National Yang-Ming University

155, Section 2, Li-Nong Street, Shipai

Taipei 112, Taiwan

Tel: +886-2-28267373

Fax: +886-2-28200565

mosquito@ym.edu.tw

Supplementary Materials and Methods

In-Gel Digestion

In-gel digestion was carried out as described by Liao *et al.* (2015)[1]. After electrophoresis, the sizes of proteins were estimated after staining with Coomassie Brilliant Blue G-250 (Bio-Rad, USA). The gel lanes were cut into ten equal fractions based on their molecular weight, and the slices were repeatedly washed in a solution of 1:125 mM NH_4HCO_3/50% (V/V) acetonitrile (ACN) until any stain was completely removed. After completely drying the gel fragments in a SpeedVac (Thermo Scientific, USA), the slices were rehydrated with 2% (V/V) β-mercaptoethanol/25 mM NH_4HCO_3 and incubated at room temperature for 20 min in the dark to reduce the disulfide bounds. Following this reduction, the slices were incubated with an equal volume of 10% (V/V) 4-vinylpyridine in 1:125 mM NH_4HCO_3/50% (V/V) ACN for 20 min to alkylate the cysteine residues. The slices were than washed by soaking in 1 mL of 25 mM NH_4HCO_3 for 10 min, dried in a SpeedVac for 20 min, and finally incubated with 100 ng of modified trypsin (Promega, Germany) in 25 mM NH_4HCO_3 37°C overnight. The tryptic digestion mixture was removed from the gel, dried in a SpeedVac and stored at -20°C.

Mass spectrometry analysis

Mass spectrometry analysis was carried out as described by Liao *et al.* (2015)[1].The tryptic peptides were resuspended in 0.1% (V/V) formic acid immediately before analysis and then separated using an online nano-acquity ultra performance liquid chromatography (UPLC) system (Waters, UK) coupled to a LTQ-Orbitrap Discovery mass spectrometer (MS) with a nano-electrospray ionization source (Thermo Scientific, USA). The peptides were separated using an Agilent C18

trapping cartridge (100 mm x 0.075 mm, particle size 3.5 μm) at a flow rate of 0.5 μL per minute in a conjunction with a gradient elution solvent system: mobile phase A was water with 0.1% formic acid and mobile phase B was ACN with 0.1% formic acid. The peptides were eluted from the column using a linear gradient of 5% to 35% of solution B for 90 min, 35% to 95% solution B for 2 min, and 95% solution B for 10 min. The eluted peptides were ionized using a spray voltage of 2 kV and then introduced into the MS. MS dataset was obtained using a data-dependent acquisition method (isolation width: 2 Da), and a full MS survey scan (m/z: 200-2,000, resolution 30,000) at half maximum width; this was followed by a tandem MS (m/z: 200-2,000) scan of the six most intense ions (doubly charged ions and triply charged charge states). Fragment ions of each of the selected precursors were generated by collision-induced dissociation using helium gas at a collision energy of 3.5 eV.

Database search

The liquid chromatography-tandem mass spectrometry raw dataset was analyzed using Xcalibur 2.0.7 SR1 (Thermo Scientific, USA), and then processed using in-house software within a Microsoft VBA environment. The resulting files were used to search against the NCBI's database (https://www.ncbi.nlm.nih.gov/) and the VectorBase website (http://www.vectorbase.org) using an in-house TurboSequest search server (ver. 27, rev. 11) (Thermo Scientific, USA). The following search parameters were incorporated: peptide mass tolerance (20 ppm); fragment ion tolerance (0.8 Da); enzyme set as trypsin; one missed cleavage allowed; and peptide charge (doubly charged ions and triply charged ions). Oxidation of methionine (+16 Da) and vinylpyridine alkylation of cysteine (+105.06 Da) were allowed as variable modifications. The TurboSequest results were filtered using criteria similar to those of

Liao *et al.* (2015)[1] and highly confident results required a DelCN value of ≤ 0.1. At least two unique peptides needed to be matched with the Xcorr score for each peptide being > 2.5. The false-discovery rate was set 1.0%.

Supplementary References

1. Liao, C. C. *et al.* Proteomics analysis to identify and characterize the molecular signatures of hepatic steatosis in ovariectomized rats as a model of postmenopausal status. *Nutrients*. **7**, 8752-8766 (2015).

Supplementary Figure

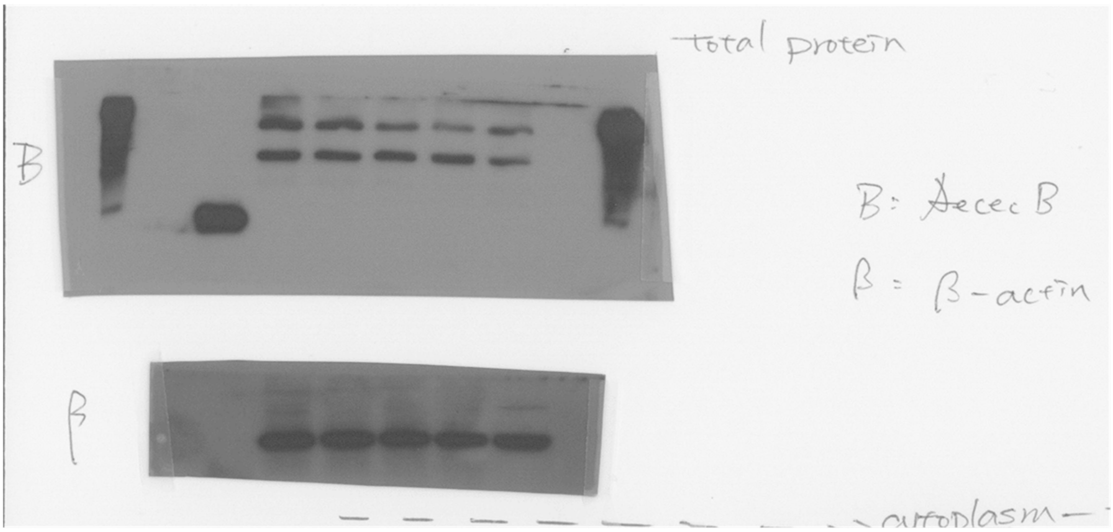

Supplementary Figure S1. Full length blots of the Western data shown in the regular Figure 1c.

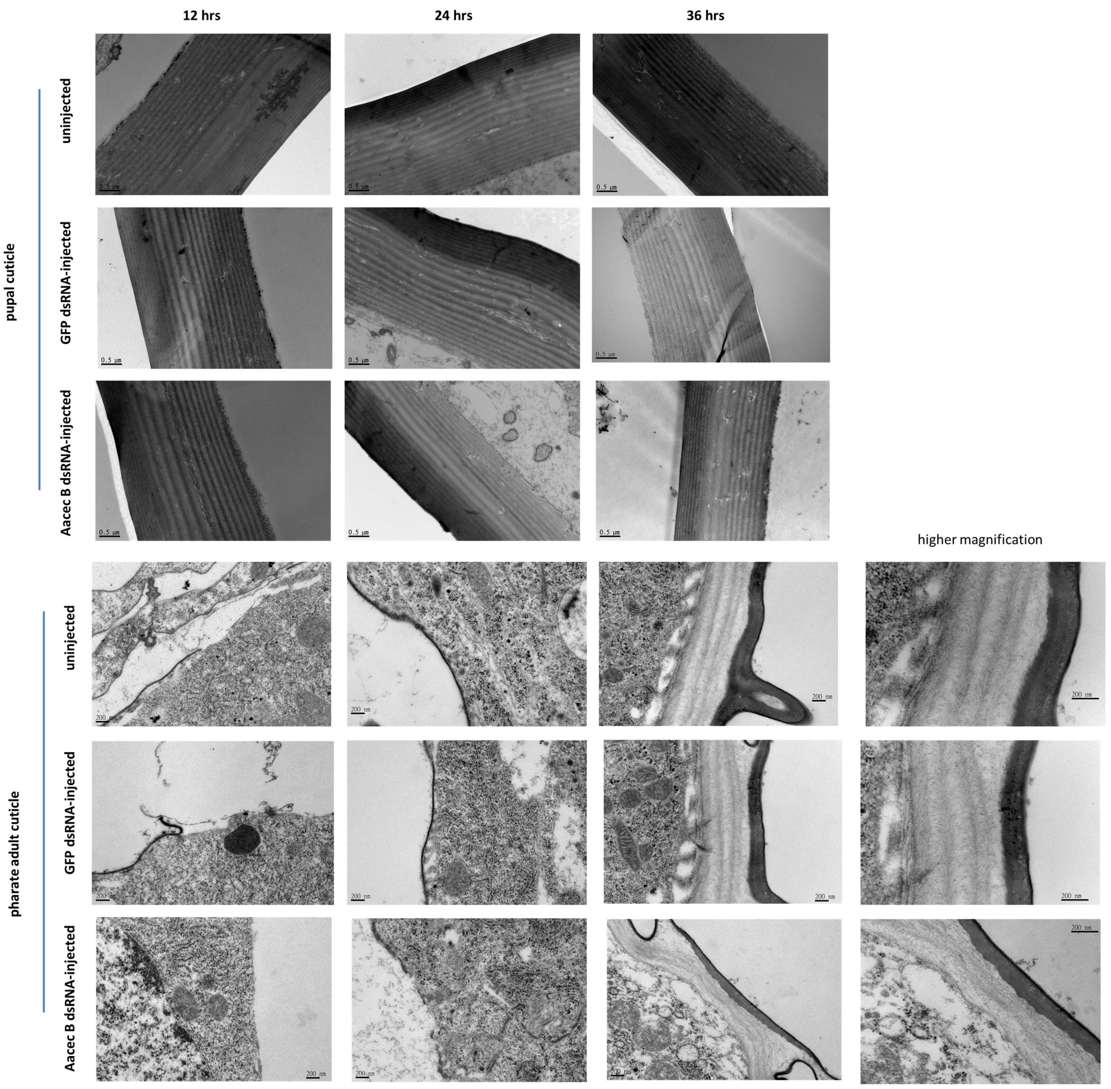

Supplementary Figure S2. Full length transmission electron micrographs of the pupal cuticle and pharate adult cuticle of the uninjected control, GFP dsRNA-injected control and Aacec B dsRNA-injected *Ae. aegypti* pupae shown in the regular Figure 3.

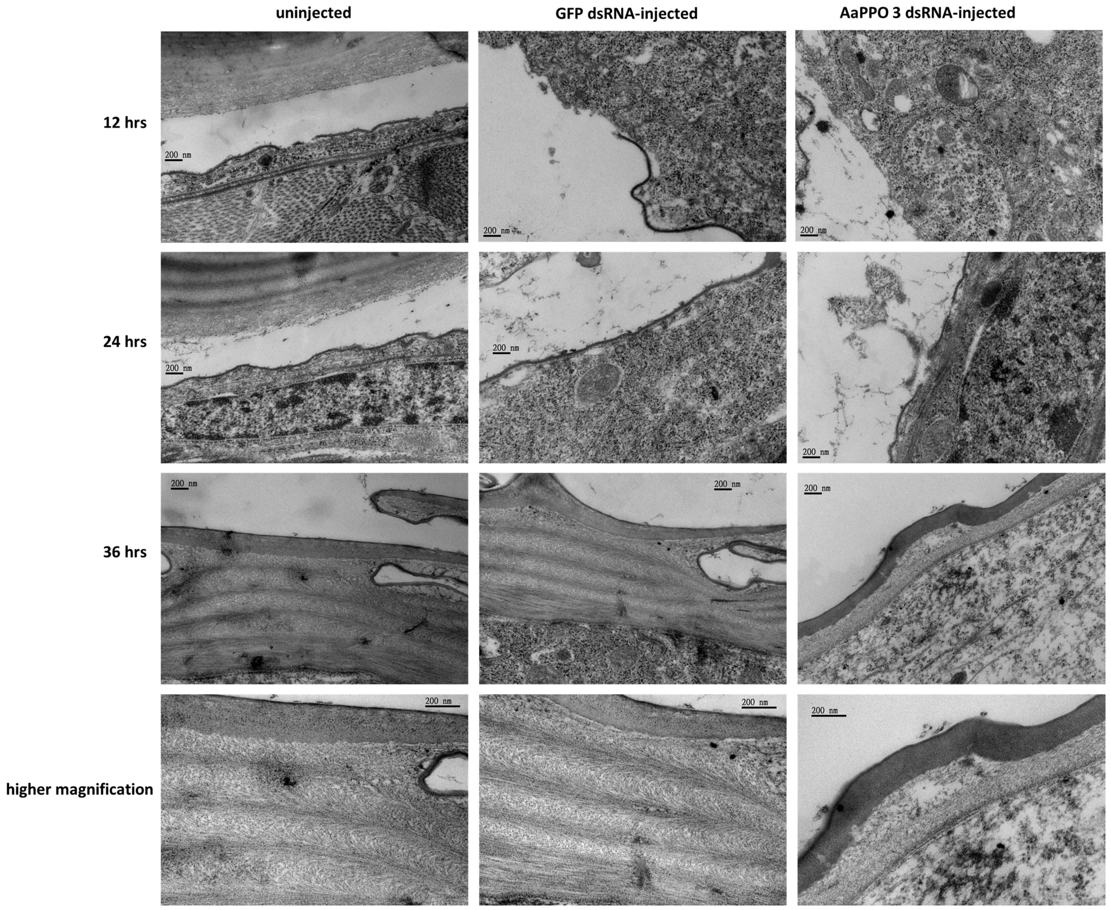

Supplementary Figure S3. Full length transmission electron micrographs of the pharate adult cuticle of the uninjected control, GFP dsRNA-injected control and AaPPO 3 dsRNA-injected *Ae. aegypti* pupae shown in the regular Figure 5e.

a

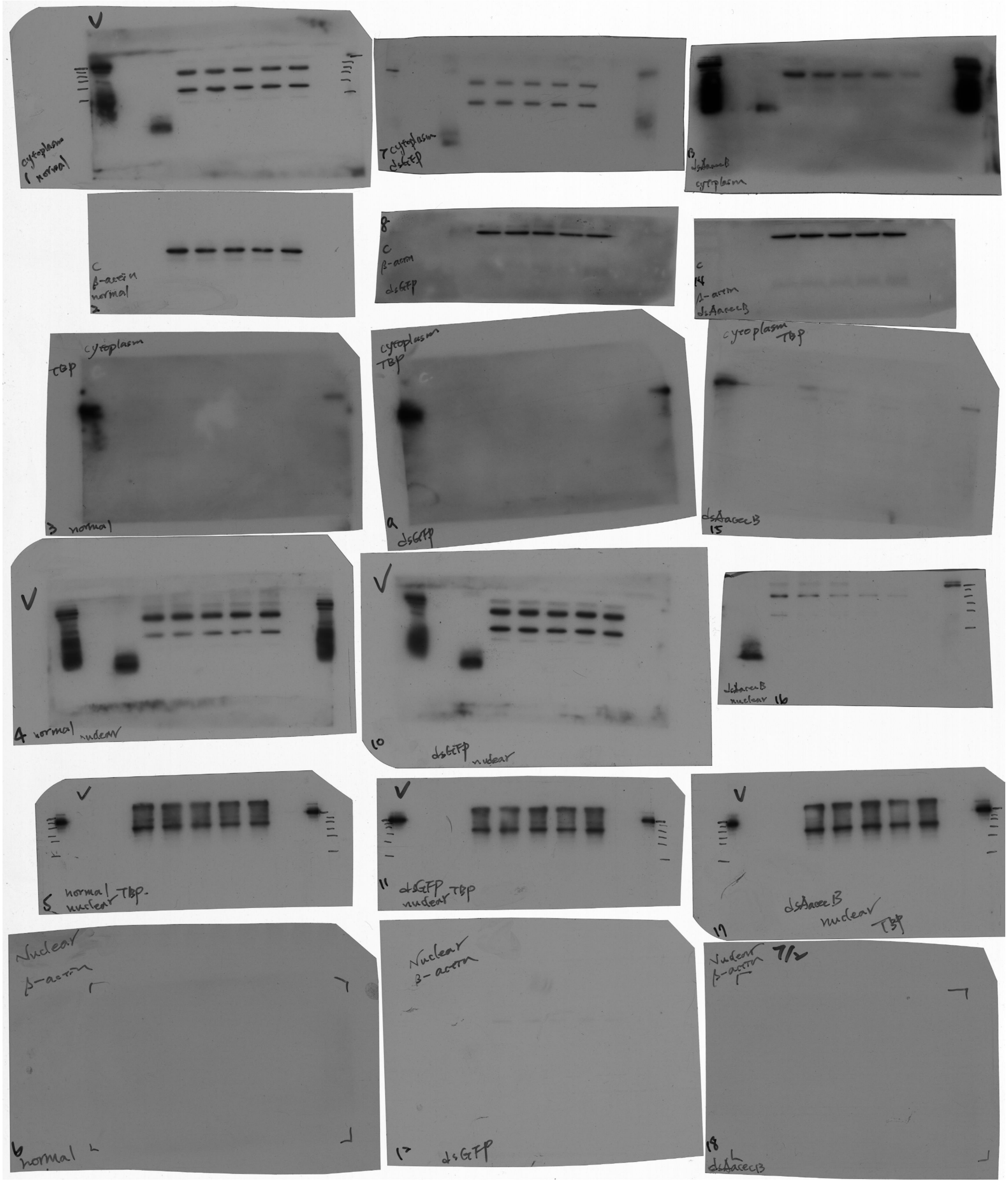

Supplementary Figure S4. Full length blots of the Western data shown in the regular Figures 6b and 6c.

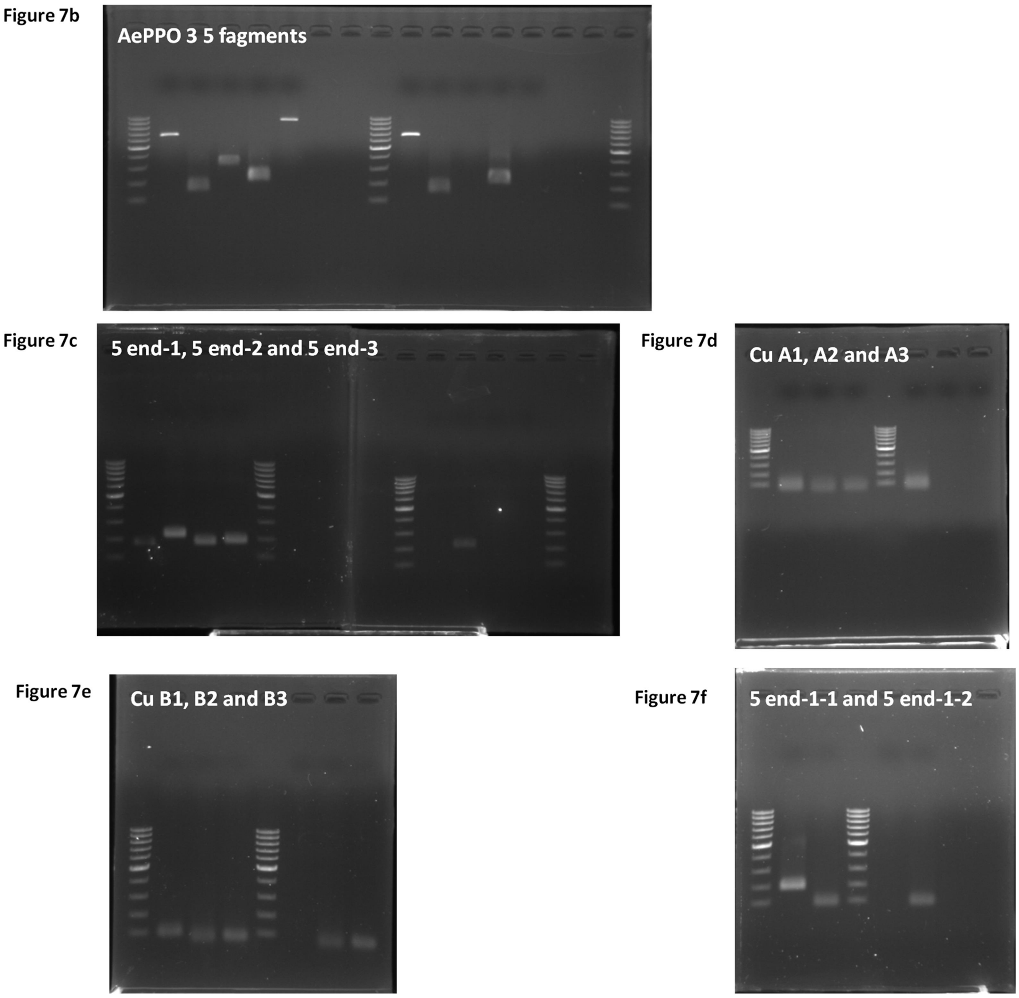

Supplementary Figure S5. Full length gels of the PCR data shown in the regular Figures 7b-7f.

a

Figure 8a

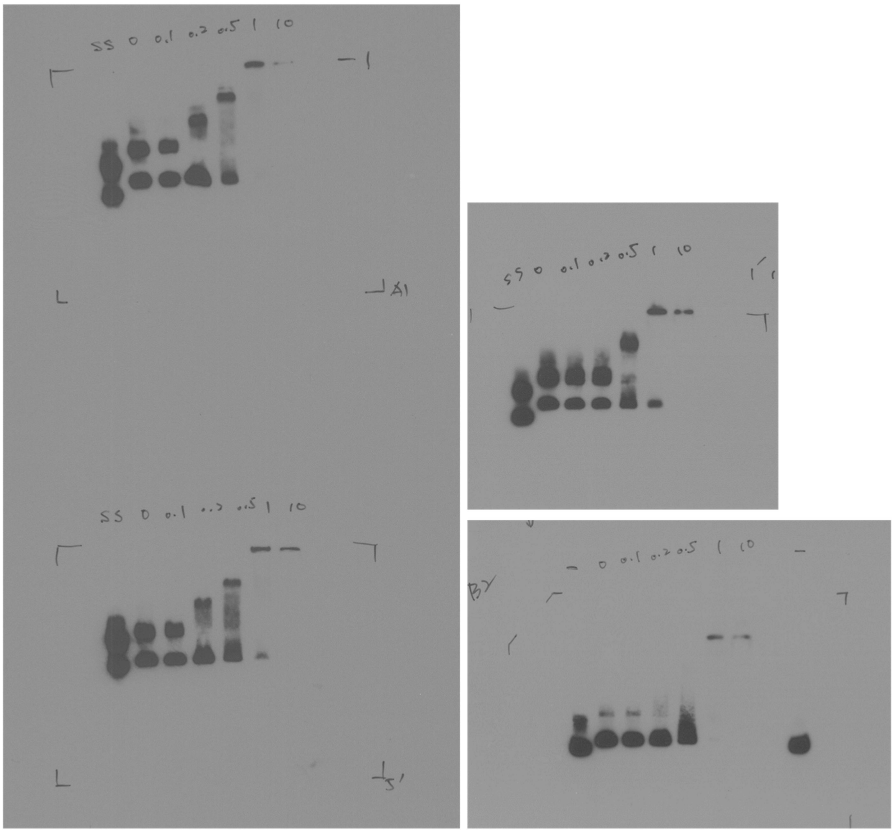

Figure 8b

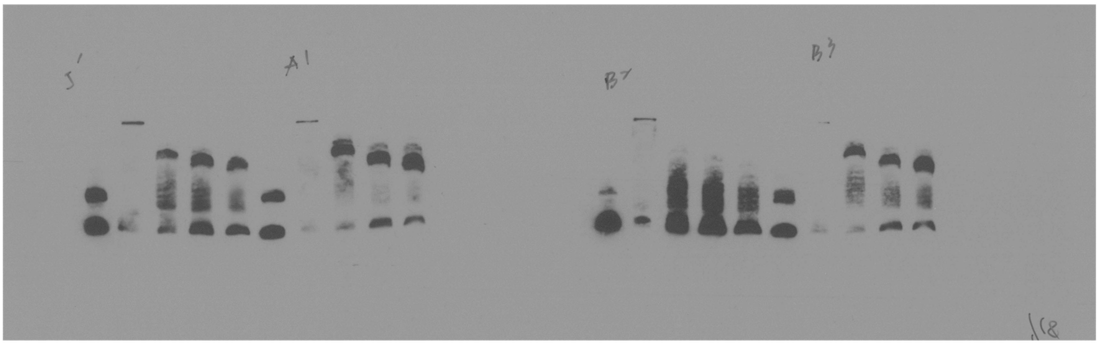

Figure 8c

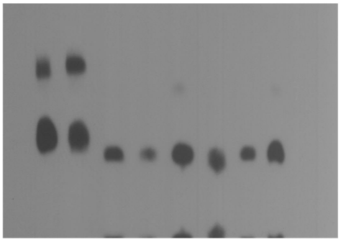

Supplementary Figure S6. (a) Full length blots of the EMSA data shown in the regular Figures 8a-8c.

b

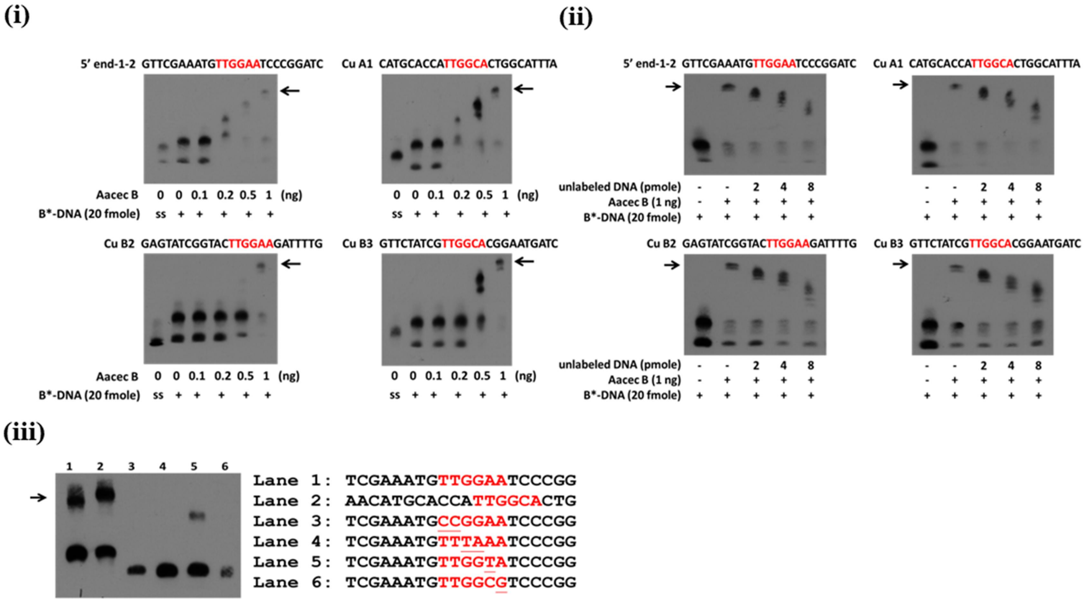

c

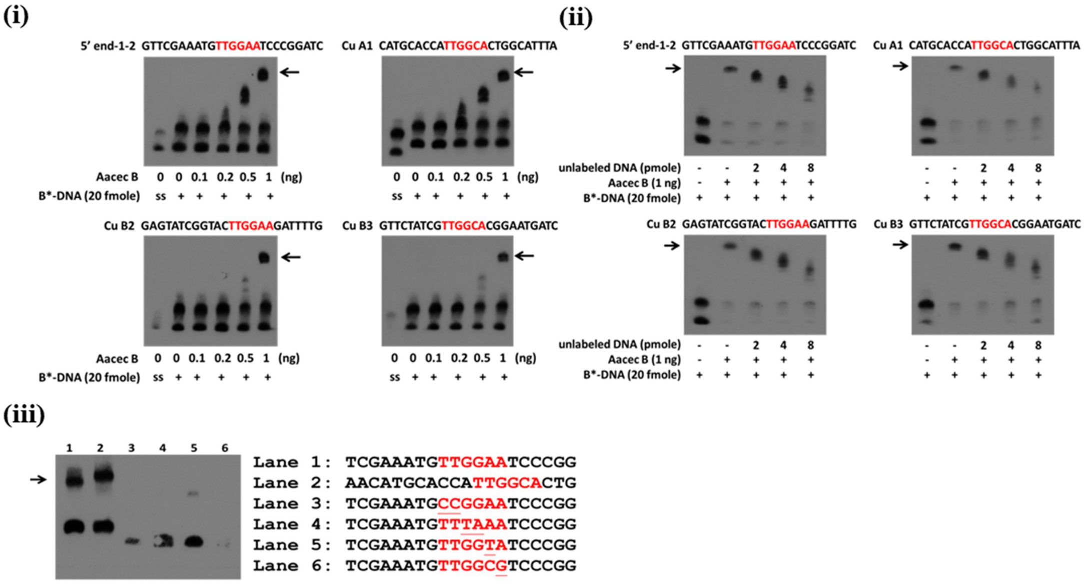

Supplementary Figure S6 continued. (b) and (c) show data from the other two biological replicates corresponding to experiments presented in Figures 8a-8c.

AePPO 1

5' end (position from +206 to +251 bp)
CCGTATTCCCGTCCGAAATGTTGGAATACCGAGTATCGCTTTCGCC

Cu A (position from +700 to +746)
ATCGGAGTCAATCTCCATCATTGGCATTGGCATTTGGTATATCCAG

Cu B (position from +1289 to +1334 bp)
ACGATCCTGAGGGTCGCTTCTTGGAAGGGTACGGAGTTGTTGGAGA

AePPO 4

Cu A (position from +688 to +733 bp)
AGGATATCGGCGTCAATCTACACCATTGGCACTGGCATCTGGTGTA

Cu B (position from +8334 to +8379 bp)
ACGATCCGGACAACTCGTTTTTGGAAAGTTTCGGAGTAGTTGGGGA

Cu B (position from +8396 to +8441 bp)
CGAGATCCAGTCTTCTACCGTTGGCACCAACACATCGACGATATTT

Supplementary Figure S7. The nucleotide sequences of the AaPPO 1 and AaPPO 4 DNA fragments containing the TTGGAA and TTGGCA putative motifs. The TTGGAA and TTGGCA putative motif are indicated in red.

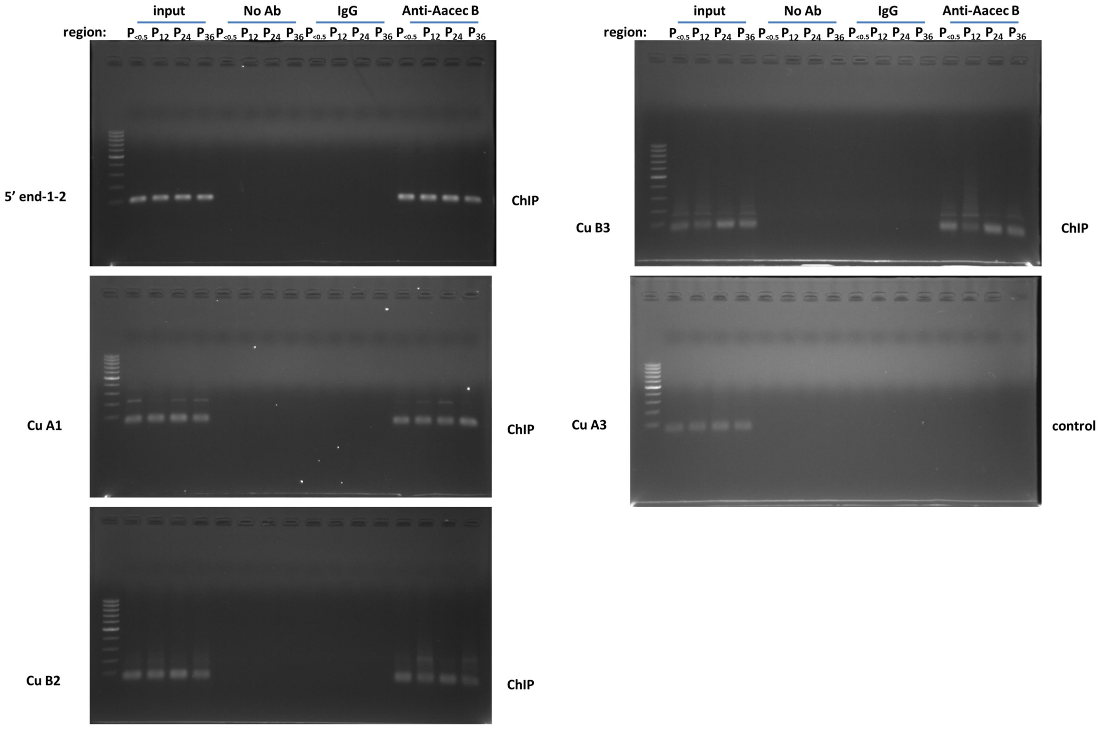

Supplementary Figure S8. Full length gels of the PCR data shown in the regular Figures 8e.

Supplementary Table

Supplementary Table S1. The number of proteins whose protein expression levels were reduced or enhanced in pupae after injection with Aacec B dsRNA

Functional categories	Reduced*	Enhanced*	Total
Information storage and processing	11	10	21
Cellular processes and signaling	9	19	28
Metabolism	48	28	76
Poorly characterized	16	3	19
Total	84	60	144

*: Compared with those in the uninjected control, the amount of each of the included proteins from the Aacec B dsRNA-injected pupae were either reduced by 2-fold or more, or enhanced by 2-fold or more.

Supplementary Table S2. A list of the thirteen cuticle formation-related proteins the protein expression of which in pupae after injection with Aacec B dsRNA was either reduced or enhanced

Protein name	Accession swissprot	MW(kDa)	Spectral counting			Theoretical isoelectric point	Protein coverage (%)	up/down regulate*
			uninjected	GFP dsRNA	Aacec B dsRNA			
AaPPO 1	Q9GYW1	78	11.836	8.384	5.789	6.59	7.89	down
AaPPO 3	Q16P46	78	1.39	0.86	0.641	6.46	1.74	down
AaPPO 4	Q174J3	79	2.612	2.286	1.238	6.67	5.38	down
chitinase II	Q17JL4	48	2.015	2.47	4.07	8.35	13.38	up
chitinase II	Q17JL7	48	1.536	1.4	3.473	6.15	19.95	up
Chitin binding protein	Q16MN2	26	1.135	1.635	2.326	4.89	23.08	up
Pupal cuticle protein 78E	Q16TU0	15	0	0.3	0.329	4.68	31.76	up
Cuticle protein	Q16VF4	26	4.979	2.501	2.475	4.93	20.76	down
Serine protease	Q17MA4	35	0.459	2.57	2.815	6.83	14.89	up
Serine protease	Q1HRH0	39	0	0.45	1.469	7.83	12.43	up
Serine protease	Q17HP8	47	0.493	0.643	1.123	5.27	7.48	up
Serine protease inhibitor, Serpin	Q179E0	50	0.739	1.15	3.03	5.45	18.57	up
Serine protease inhibitor, Serpin	Q16HB8	52	0	0.3	0.329	5.46	7.17	up

*: Compared with those in uninjected pupae, the amount of protein was increased or decreased in the Aacec B dsRNA-injected pupae.

Supplementary Table S3. Specific primer pairs used for RT-qPCR

Primer name	Accession number	Primer sequence
Aacec A-F	AAEL000627	ATTTCTCCTgATCgCCgTggCTg
Aacec A-R		gAgCCTTCTCggCggCATTgAA
Aacec B-F	AAEL004223	TTggTgCCACTATTCgAggA
Aacec B-R		gAgCgCTgCCAggATAACAA
Aacec D-F	AAEL000598	gCTAggTCAAACCgAAgCAg
Aacec D-R		TCCTACAACAACCgggAgAg
Aacec E-F	AAEL000611	CgAAgCCggTggTCTgAAg
Aacec E-R		ACTACgggAAgTgCTTTCTCA
Aacec F-F	AAEL000625	gTgTTCAAAgCATCggAAAAAg
Aacec F-R		gCTgACATTCACAATCTATCTCCg
Aacec G-F	AAEL015515	TCACAAAgTTATTTCTCCTgATCg
Aacec G-R		gCTTTAgCCCCAgCTACAAC
Aacec H-F	AAEL017211	CTTCACCAAgCTgCTATTggT
Aacec H-R		AACTTTTTTgCCAATCTTCTTCAgC
Aacec I-F	AAEL000775	ggCTATTCgTTTTCgTCATTTTC
Aacec I-R		CTACgTTTTTgCCAgCCTTTTC
Aacec J-F	AAEL000777	gCTATTCgTTTTCgTCATTTTTg
Aacec J-R		CTTTTCAATCTTTTTgCCCAg
Aacec N-F	AAEL000621	CggCAAgAAATTggAAAAAgTC
Aacec N-R		gAATCgATCATCCTAgggCC
AaS7-F	XM_001660119	gCTgAAgTCgTCggCAAgCgTAT
AaS7-R		CgTCACgTCCggTCAgCTTCTT
AaPPO 1-F	AAEL013498	gCATTgCTgCAgCgTCCTTT
AaPPO 1-R		gggTCTCTgCATCgTCACTgAA
AaPPO 3-F	AAEL011763	ATCTTTCCAAgCgCTTCTgCAACg
AaPPO 3-R		ATTTCgACCTCCgCgCCAC
AaPPO 4-F	AAEL013501	ACCCAggCgAgAATAATgTAgTgC
AaPPO 4-R		CgCTCTCgggTTgATTggAC

qPCR conditions: 1. An initial step: 95°C for 3 min, 2. 40 cycles at 95°C for 15 s and 54°C for 30 s.

Supplementary Table S4. Specific primer pairs used for RT-qPCR

Primer name	Accession number	Accession swisprot	Primer sequence
Chitin binding protein-F	XM_001662303	Q16MN2	TgAAgTgTTTggTggTgTgTCg
Chitin binding protein-R			CTTCACACTTCACTCCACTCgAgTC
Pupal cuticle protein 78E-F	XM_001660599	Q16TU0	AgCACAggTCTgATTCTgATgg
Pupal cuticle protein 78E-R			CgTTgCTCgTTTCgTAggTA
Cuticle protein-F	XM_001660199	Q16VF4	gTCAACTTTTgggCTgTCTggg
Cuticle protein-R			CCgCAAACACggCAATTAgAAC
Serine protease-F	XM_001647815	Q1HRH0	ggTCgTTCTgAACgATTCgggATTC
Serine protease-R			TgCACCCAACgTTgCCAATC
Serpin-F	XM_001651181	Q179E0	ACTggAgATTgggCgAAgCA
Serpin-R			gCTgTCCTCgAAATggCTgAT

qPCR conditions: 1. An initial step: 95°C for 3 min, 2. 40 cycles at 95°C for 15 s and 54°C for 30 s.

Supplementary Table S5. Specific primer pairs used for PCR

Primer name	Primer sequence
AaPPO 3 5' end--56-F	TCTAATggTCACATATAAAAg
AaPPO 3 5' end-1-F	CCAATCTCCAATCgATAgCT
AaPPO 3 5' end-689-R	CTCTgTTCTggTTCgCgATC
AaPPO3-5' end-55-F	ATggCTgAgAgTAAATCTTTCCAAg
AaPPO3-5' end-157-R	ACTCgTCCggAATTATgAgCgATgT
AaPPO3 5' end-274-F	gTTATCgATCgTCgTggAgC
AaPPO3 5' end-549-R	CCgATgCTggATTgCCACCg
AaPPO3 5' end-460-F	CCAgACACTAAAgATgTACCgATT
AaPPO3 5' end-690-R	TCTCTgTTCTggTTCgCgAT
AaPPO3-5' end-180-F	gTTAgCTggCgATTTgCAgAgCAgg
AaPPO3-5' end-183-R	TAACggACgATAACgATCAC
AaPPO3-5' end-273-R	CTCCgCAAAggCAAgATCCg
AaPPO 3 Cu A-691-F	ATggCATACTTCCgTgAggA
AaPPO 3 Cu A-840-R	ATAACgAgAAATgATTTggC
AaPPO 3 Cu A1-749-R	ACTAAATgCCAgTgCCAATg
AaPPO 3 Cu A2-750-F	gTATCCTgCTggggCgCCAC
AaPPO 3 Cu A2-791-R	CgATCTTTCTTAgCAATTTC
AaPPO 3 Cu A3-792-F	CCgTggTgAACTTTTCTACT
AaPPO 3 Cu A3-840-R	ATAACgAgAAATgATTTggC
AaPPO 3 inter-Cu-841-F	AATgTggATCgATTCTgTAA
AaPPO 3 inter-Cu-1189-R	gTCgATTCCTTTgACTTCAT
AaPPO 3 Cu B-1190-F	ATTTTAggTgACTTggTTgAAg
AaPPO 3 Cu B-1395-R	AACAAgCCATCgATCATTCC
AaPPO 3 Cu B1-1276-R	CAAAAAATCATgTCCAAAgT
AaPPO 3 Cu B2-1277-F	gCCTACATTCACgATCCTgA
AaPPO 3 Cu B2-1329-R	CCCATTACTCCAAAATCTTC
AaPPO 3 Cu B3-1330-F	TgACgTgACAACggCAATgC
AaPPO 3 Cu B3-1394-R	ACAAgCCATCgATCATTCC
AaPPO 3 3' end-1415-F	CTAAACCCATACACTTCTAC
AaPPO 3 3' end-2563-R	CCgCATTCACCATTTATggCAgA
AaPPO 3 5' end-235-F	gTTCgAAATgTTggAATCCCggATC
AaPPO 3 5' end-259-R	gATCCgggATTCCAACATTTCgAAC
AaPPO 3 Cu A1-723-F	CATgCACCATTggCACTggCATTTA
AaPPO 3 Cu A1-747-R	TAAATgCCAgTgCCAATggTgCATg
AaPPO 3 Cu B2-1295-F	gAgTATCggTACTTggAAgATTTTg
AaPPO 3 Cu B2-1319-R	CAAAATCTTCCAAgTACCgATACTC
AaPPO 3 Cu B3-1360-F	gTTCTATCgTTggCACggAATgATC
AaPPO 3 Cu B3-1384-R	gATCATTCCgTgCCAACgATAgAAC

PCR conditions: 94°C for 30 s, 53°C for 30 s, and 72°C for 30 s for 26 cycles with a final extension at 72°C for 2 min.

Supplementary Table S6. The specific single-stranded oligonucleotides used for EMSA and qChIP assay.

name	DNA sequence
AaPPO 3-5’ end-1-2 F	GTTCGAAATGTTGGAATCCCGGATC
AaPPO 3-5’ end-1-2 R	GATCCGGGATTCCAACATTTCGAAC
AaPPO 3-Cu A1 F	CATGCACCATTGGCACTGGCATTTA
AaPPO 3-Cu A1 R	TAAATGCCAGTGCCAATGGTGCATG
AaPPO 3-Cu B2 F	GAGTATCGGTACTTGGAAGATTTTG
AaPPO 3-Cu B2 R	CAAAATCTTCCAAGTACCGATACTC
AaPPO 3-Cu B3 F	GTTCTATCGTTGGCACGGAATGATC
AaPPO 3-Cu B3 R	GATCATTCCGTGCCAACGATAGAAC
TTGGCA-nucleotide replacement 1-F	TCGAAATGCCGGAATCCCGG
TTGGCA-nucleotide replacement 1-R	CCGGGATTCCGGCATTTCGA
TTGGCA-nucleotide replacement 2-F	TCGAAATGTTTAAATCCCGG
TTGGCA-nucleotide replacement 2-R	CCGGGATTTAAACATTTCGA
TTGGCA-nucleotide replacement 3-F	TCGAAATGTTGGTATCCCGG
TTGGCA-nucleotide replacement 3-R	CCGGGATACCAACATTTCGA
TTGGCA-nucleotide replacement 4-F	TCGAAATGTTGGCGTCCCGG
TTGGCA-nucleotide replacement 4-R	CCGGGACGCCAACATTTCGA

qPCR conditions: 1. An initial step: 95°C for 3 min, 2. 40 cycles at 95°C for 15 s and 54°C for 30 s.
